# Personalized Feedback Interventions for Indicated Prevention of Gambling Disorder: A Systematic Review and Meta-Analysis

**DOI:** 10.1007/s10899-025-10444-5

**Published:** 2025-10-29

**Authors:** E. Halle Smith, Bre’Anna L. Free, Meredith K. Ginley, James P. Whelan, Rory A. Pfund

**Affiliations:** 1 Tennessee Institute for Gambling Education & Research, Memphis, TN USA; 2 Department of Psychology, University of Memphis, Memphis, TN USA; 3 Department of Psychology, East Tennessee State University, Johnson City, TN USA

**Keywords:** Gambling, Brief intervention, Normative feedback, Gambling harm

## Abstract

While personalized feedback interventions (PFIs) are a promising indicated prevention strategy for reducing gambling behavior and gambling harm immediately following intervention, no meta-analysis has examined the durability of these reductions. The current systematic review and meta-analysis aimed to examine the effect of PFIs immediately following the intervention and in the months following the intervention. The Cochrane Central Register of Controlled Trials, Embase, PsycINFO, PubMed were searched in January 2024 to identify randomized controlled trials comparing PFIs to standard care or inactive control conditions. The Cochrane Risk of Bias tool version 2.0 assessed risk of bias. Correlated, hierarchical random effect meta-analysis was conducted to examine the effect of PFIs at postintervention and follow-up. Eighteen studies representing 9,869 participants were identified. PFIs had nonsignificant, small reductions in outcomes relative to comparison conditions at postintervention (*g* = -0.06, 95% CI [-0.15, 0.02]). However, PFIs produced significant, small reductions in outcomes relative to comparison conditions at follow up (*g* = -0.10, 95% CI [-0.18, -0.02]). Therapist facilitated PFIs (*g* = -0.18) had significantly better outcomes than non-therapist facilitated PFIs (*g* = -0.03), and PFIs incorporating motivational interviewing (*g* = -0.19) had significantly better outcomes than PFIs not incorporating motivational interviewing (*g* = -0.02). However, there was limited statistical power to estimate effect sizes at precise time intervals over the follow-up period. The field requires more randomized controlled trials with longitudinal assessments to estimate the effectiveness of PFIs for gambling in the months following receipt of the intervention. PROSPERO (CRD42024567384).

**Supplementary Information:**

The online version contains supplementary material available at 10.1007/s10899-025-10444-5.

Gambling, or risking something of monetary value on an event that is at least partially determined by chance (Whelan et al., 2007), is a common recreational activity across the world. Approximately 46% of adults gambled at least once in the past year across a number of different gambling activities (Tran et al., 2024). Furthermore, the value of the global gambling market was estimated at $474 billion in 2023 (Statista, 2025), and the global gambling market value is expected to increase to $700 billion in 2028 (H2 Gambling Capital, 2024).

As technology has evolved and the legalization of gambling has expanded across jurisdictions, concerns have mounted about gambling harm (Ukhova & Volberg, 2025). Approximately 9% of adults engage in risk gambling (Tran et al., 2024) that involves experiencing gambling harm such as financial problems, interpersonal conflicts, reduced performance at work/school, and/or psychological distress that has transient or intergenerational consequences (Langham et al., 2016). Furthermore, individuals experiencing gambling harm negatively affect at least six other people (Goodwin et al., 2017). The collective harms from gambling, its indirect negative effects on other people, and loose government regulations have made the issue of preventing gambling harm more important than ever (Wardle et al., 2024).

Personalized feedback interventions (PFIs) are a secondary prevention strategy that aim to reduce gambling behavior and gambling harm. PFIs generally involve collecting information from individuals about their gambling behaviors and gambling harm and providing them with feedback about how frequently they gamble, how much they gamble, how long they gamble, whether they are experiencing gambling harm, and/or the extent of that harm. PFIs vary in how this information is presented, as some provide feedback based on normative comparisons (i.e., comparing behaviors to a relevant reference group; Neighbors et al., 2015) and others integrate motivational interviewing principles to encourage individuals to generate values-based reasons to change their gambling after receiving feedback (Larimer et al., 2012; Petry et al., 2009).

A meta-analysis on randomized controlled trials found that PFIs reduced gambling behavior and gambling harm (*d* = 0.20, 95% CI [0.12, 0.27]) relative to standard care control conditions (Peter et al., 2019). However, the previous meta-analysis has several conceptual and methodological limitations. First, the meta-analysis calculated the effect size based on studies that combined cognitive-behavioral workbooks with PFIs (Hodgins et al., 2001; Hodgins, Currie, Hodgins et al., 2009a, b). Given that cognitive-behavioral interventions, including workbooks, have been found efficacious for reducing gambling behavior and gambling problems relative to no intervention (Pfund, Forman, Pfund et al., 2023a, b, c; Pfund, Ginley, Pfund et al., 2023a, b, c; Pfund, King, Pfund et al., 2023a, b, c), it is unclear whether the effect size estimate was due to PFIs or the cognitive-behavioral workbooks. The inclusion of cognitive-behavioral interventions could unintentionally inflate the effect size.

Second, the meta-analysis did not separate outcomes into specific domains of gambling behavior (e.g., gambling frequency versus expenditure) or gambling harm (e.g., financial difficulties, interpersonal conflict). The combination of outcomes is problematic, as the intervention may affect some outcomes more than others (Pfund et al., 2023). Some PFIs target only gambling expenditures (Jonsson et al., 2019) and do not extend feedback to other dimensions of gambling behavior, such as how frequently someone is gambling or the severity of their gambling problems (Martens et al., 2015). Without targeting specific gambling behaviors or gambling harm, it is unclear whether PFIs affect all outcomes equally.

Third, the meta-analysis did not examine the durability of outcomes across time. The meta-analysis focused on “short-term effects… from whichever follow-up time-point was closest to 1-month postintervention” (p. 454). This methodological approach focuses on understanding the short-term effects of PFIs and prohibits understanding whether effects are maintained over time. Meta-analyses on cognitive-behavioral treatments have found that the magnitude of effect size estimates decreases over time (Pfund et al., 2023; Pfund, Ginley, Pfund et al., 2023a, b, c), and it is possible that the same is true of PFIs.

The aim of the current systematic review and meta-analysis was to understand the effect of PFIs on gambling behaviors and harm over time. This meta-analysis was distinct from the previous meta-analysis in that it excluded studies if they combined PFIs with any cognitive-behavioral treatment, analyzed outcomes (i.e., gambling frequency, expenditure, duration, and harm) separately, and examined the effects of PFIs on outcomes across time. Thus, the current meta-analysis expands foundational work to more precisely understand the effect of PFIs on gambling outcomes as a secondary prevention strategy.

## Method

The following systematic review and meta-analysis were preregistered on PROSPERO (CRD42024567384). However, there were three notable deviations from preregistration. The first deviation was that the current systematic review and meta-analysis did not include tertiary prevention studies that involved individuals with gambling disorder. After conducting the systematic article search, no tertiary prevention studies were identified and were therefore excluded from the review. The second deviation was that we reconceptualized one of the moderator variables, “cutoff for inclusion,” that categorized individuals as either “clinical” or “subclinical” samples, as was done in the Peter et al. (2019) meta-analysis. However, this moderator variable was recategorized because there were no clinical samples/tertiary prevention studies. The new variable was categorized into two groups representing studies that required participants to experience gambling harm to be eligible for the study or studies that did not require participants to experience gambling harm to be eligible for the study. The third deviation was that an additional moderator was included as an exploratory variable to examine differences in outcomes based on the presence of normative feedback versus not.

The method was consistent with the Preferred Reporting Items for Systematic Reviews and Meta-Analyses (Page et al., 2021) and a Measurement Tool to Assess Systematic Reviews-2 (Shea et al., 2017) guidelines.

### Search Strategy

Four bibliographic databases were initially searched in January 2024 and then updated in January 2025. To identify additional articles for inclusion, reference lists of two past meta-analyses on PFIs for gambling were also searched (Peter et al., 2019; Saxton et al., 2021). The search had no start date or language restrictions, and grey literature was eligible. Supplemental Table 1 contains a list of databases searched and the search strings for each database.

### Inclusion and Exclusion Criteria

Studies were included if they involved (a) participants aged 18 years or older, (b) participants who gambled and/or experienced subclinical gambling harm, (c) an intervention described as providing feedback to individuals about any dimension of their gambling behavior (e.g., gambling frequency, gambling expenditure, gambling duration) or gambling problem severity, (d) a randomized controlled trial design with random assignment to two or more study conditions, and (e) standard care (i.e., psychoeducation) or inactive (waitlist, assessment only) comparison conditions as the control group. Studies were excluded if they involved (a) planned study protocols with no published outcomes, (b) participants taking pharmacotherapy for gambling as part of their medical care or unrelated to the study, as it is possible that pharmacotherapy may affect outcomes (Dowling et al., 2022), and (c) included participants who had gambling disorder, as these studies represent PFIs in the context of tertiary prevention.

### Study Identification & Data Extraction

Publications from the bibliographic database search were imported into the Covidence software (Veritas Health Innovation, 2024). Two authors identified studies for eligibility at both the abstract/title level and then the full-text level. Discrepancies were resolved through discussion with a third author. Supplemental Table 2 displays all the articles reviewed at the full-text level and the primary reason for exclusion.

The outcomes were gambling behavior and gambling harm, which were selected based on recommendations from the Banff, Alberta Consensus (Walker et al., 2006). Gambling behavior outcomes included gambling frequency (e.g., the number of days gambled), gambling expenditure (e.g., the amount of money spent on gambling), and gambling duration (e.g., the number of hours gambled). Gambling harm was defined as continuous measures of gambling problems based on an assessment with some reliability and validity support.

All outcomes were extracted at postintervention and follow-up in duplicate. Postintervention represented the first available assessment immediately following the PFI to replicate what was done in the Peter and colleagues (2019) meta-analysis, and follow-up represented the assessment the farthest from the PFI (Pfund et al., 2023). Time points were reported in weeks since the completion of the PFI, and studies reporting time in months were converted to weeks by multiplying the number of months by four. Sensitivity analyses were conducted to understand differences based on specific outcomes and other time points.

Moderators were extracted to replicate those in Peter and colleagues’ (2019) meta-analysis. Specifically, the moderators were (1) recruitment from a community sample (yes/no), (2) the presence of therapist facilitating the PFI (yes/no); (3) the presence of motivational interviewing principles in the PFI (yes/no); (4) the inclusion of feedback about an individual’s measured gambling harm severity (yes/no); (5) the presence of psychoeducational material about gambling (yes/no), and (6) the requirement for participants to screen positive for experiences of gambling harm for eligibility in the study (yes/no). Additionally, the presence of normative feedback (yes/no) was assessed as a possible moderator.

### Risk of Bias Assessment

Two authors independently rated all studies using the Cochrane Risk of Bias tool 2.0 (Higgins & Green, 2008). Bias was assessed in the randomization process, deviations in intended interventions, the completeness of outcome data, the appropriateness of outcome measurement, and the selection of reported results. Each domain was categorized as low risk, high risk, or some concerns. Discrepancies in ratings were resolved through discussion.

### Data Analytic Plan

Analyses were conducted using the “meta,” “metafor,” “robumeta,” and “clubSandwich” packages in R version 4.2.1. The metafor package was used to calculate Hedges’s *g* values representing between-group differences in outcomes for the PFI and control condition at postintervention and follow-up. The robumeta and clubSandwich packages were used to estimate pooled effect sizes based on correlated, hierarchical effects meta-regression models with robust variance estimates. A hierarchical effects model was used to account for dependency in multiple PFI conditions within each study, and a correlated effects model was used to account for dependency in multiple effect sizes reported in each study. The value of ρ was assumed 0.6 because no value has been empirically estimated using individual patient data meta-analysis. Analyses were conducted to examine differences in Hedges’s *g* effect sizes at postintervention and the longest available follow-up where all outcomes were combined. Results were only presented for models with adequate degrees of freedom after accounting for small sample sizes (Tipton, 2015). Outcomes were also examined separately to compare the effect of PFIs on each outcome domain (i.e., gambling frequency, expenditure, duration, and harm). When more than one assessment of the same outcome was included in the same study, the assessment that was most common among the other studies was selected.

Heterogeneity was assessed using multiple indicators, including Cochran’s *Q*, *I*^2^, and 95% prediction intervals (PI). The “meta” package was used to conduct subgroup analyses that examined differences in Hedges’s *g* values based on the six categorical moderators.

## Results

### Study Identification

**Fig. 1:**
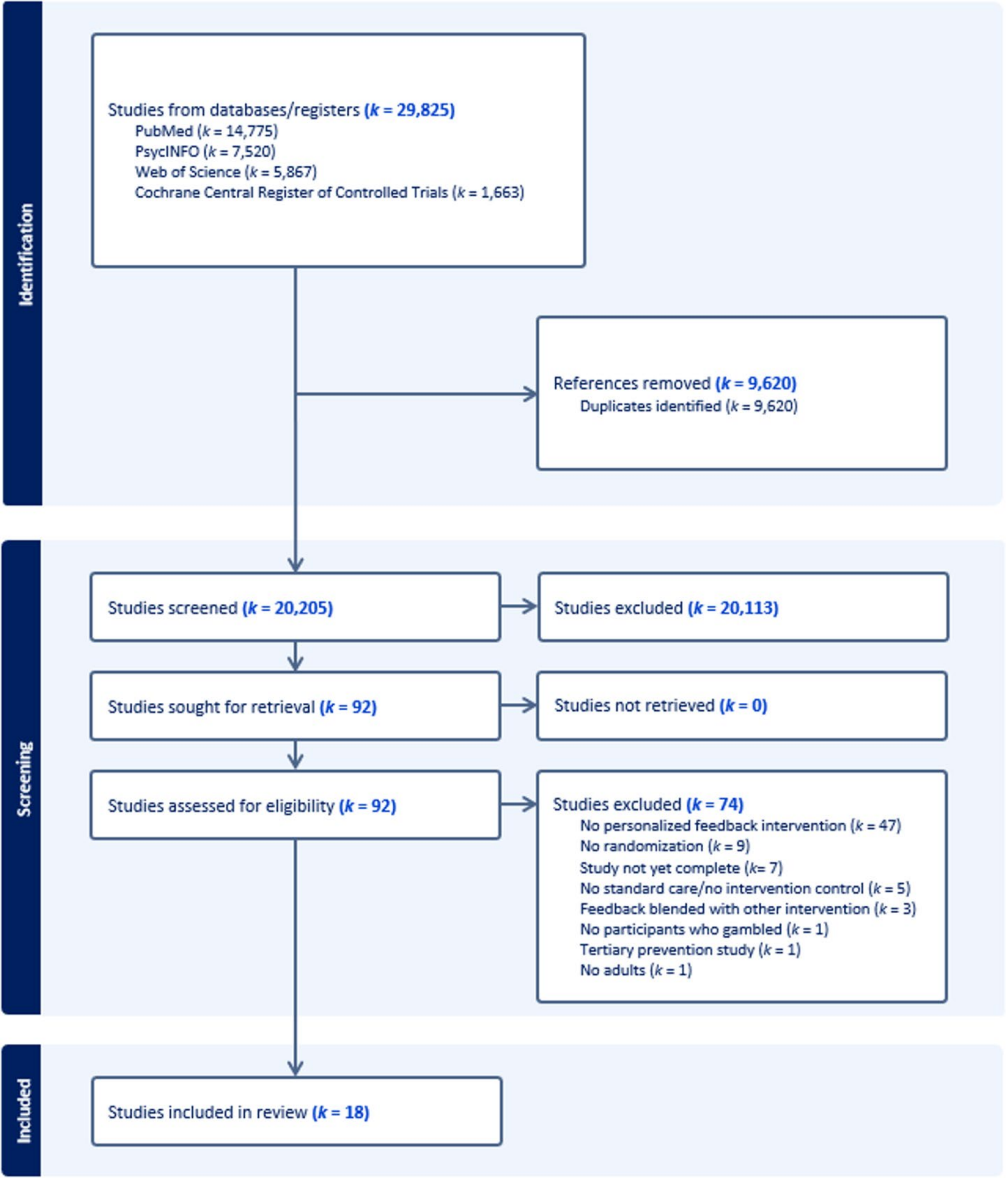
Flowchart of Studies Included in the Systematic Review and Meta-Analysis

A flowchart of the study identification process is displayed in Fig. 1. After the removal of 9,620 duplicates, 92 studies were eligible for full-text review. Eighteen studies met the inclusion criteria for the systematic review and meta-analysis. Nine of the studies (50%) were included in the previous systematic review and meta-analysis of PFIs for gambling (Peter et al., 2019), and nine studies (50%) were unique to the present systematic review and meta-analysis.

### Study, Sample, and Intervention Characteristics

Table 1 presents study characteristics for all 18 studies identified for inclusion in the systematic review and meta-analysis. Across the 18 studies, years of publication ranged from 2008 to 2024. Most studies were conducted in the United States of America (67%) or Canada (17%). Thirteen studies were grant-funded (72%), and six (33%) were preregistered.

**Table 1:** Studies included in the systematic review and meta-analysis on personalized feedback interventions for gambling

Study	Country	Funding Source	Pre-registered?	Sample recruited	Participant eligibility criteria
Broussard and Wulfert (2019)	USA	None/NR	N	Community	SOGS ≥ 2, gamble primarily on scratch-off tickets, and spend at least $10 biweekly on scratch-off tickets in past 3 months
Celio and Lisman (2014)	USA	American Psychological Association Dissertation Research Award, National Institute on Alcohol Abuse and Alcoholism	N	College	Gambled in past 30 days
Cunningham et al. (2009)	Canada	Ontario Problem Gambling Research Centre	N	Community	DSM-IV gambling dependence
Cunningham et al. (2012)	Canada	Ontario Problem Gambling Research Centre	Y	Community	PGSI ≥ 3
Hopper (2008)	USA	None/NR	N	College	Gambled twice in past 30 days
Jonsson et al. (2019)	Sweden	None/NR	N	Gambling website users	Top 0.5% of individuals who lost money
Larimer et al. (2012)	USA	National Institute on Mental Health, National Institute on Drug Abuse, National Institute on Alcohol Abuse and Alcoholism	Y	College	SOGS > 3
Lostutter (2009)	USA	National Institute on Drug Abuse	N	College	SOGS > 3
Luquiens et al. (2016)	France	Winamax	Y	Community	PGSI ≥ 5
Martens et al. (2015)	USA	National Center for Responsible Gambling	N	College	Gambled in past 30 days, SOGS ≥ 3 or BBGS ≥ 1
McAfee et al. (2020)	USA	International Center for Responsible Gaming	N	College	Gambled in past 60 days, SOGS ≥ 3 or BBGS ≥ 1
Neighbors et al. (2015)	USA	National Center for Responsible Gambling	N	College	SOGS ≥ 2
Petry et al. (2008)	USA	Patrick and Catherine Weldon Donaghue Medical Research, National Institutes of Health Grants	N	Community	SOGS ≥ 3, gambled 4 times in past 60 days, wagered at least $100
Petry et al. (2009)	USA	Patrick and Catherine Weldon Donaghue Medical Research, National Institutes of Health	Y	College	SOGS ≥ 3, gambled 4 times in past 60 days, wagered at least $100
Petry et al. (2016)	USA	None/NR	Y	Substance use treatment center	SOGS ≥ 3, gambled 4 times in past 60 days, wagered at least $100
Rosen et al. (2020)	USA	None/NR	N	Probation and parole office	PGSI ≥ 3
Tabri et al. (2024)	Canada	International Center for Responsible Gaming	Y	Crowdsourcing website	Gambling in past 3 months
Yokomitsu et al. (2024)	Japan	Ministry of Health, Labour, and Welfare	N	Crowdsourcing website	PGSI ≥ 3

*NR* not reported, *USA* United States of America, *DSM-IV* Fourth Edition of the Diagnostic and Statistical Manual of Mental Disorders, *N *no, *PFI* personalized feedback intervention, *PGSI* Problem Gambling Severity Index, *Y* yes, *SOGS* South Oaks Gambling Screen

The specific settings where participants were recruited for the studies varied widely. Most studies recruited participants from colleges (44%) and the community (28%). Other studies recruited samples from a gambling website (Jonsson et al., 2019), an inpatient substance use treatment center (Petry et al., 2016), probation and parole offices (Rosen et al., 2020), and crowdsourcing websites (Tabri et al., 2024; Yokomitsu et al., 2024).

The assessments of gambling harm and gambling behavior used to determine participants’ eligibility in the studies also varied widely. Eight studies (44%) relied solely on an assessment of gambling harm, six (33%) used a combination of an assessment of harm and some gambling behavior, and four (23%) relied solely on an assessment of gambling behavior. Of the 14 studies using assessments of gambling harm to determine participant eligibility, the most common assessments were the South Oaks Gambling Screen and the Problem Gambling Severity Index. Other assessments included the Brief Biosocial Gambling Screen or “gambling dependence” based on the Diagnostic and Statistical Manual of Mental Disorders (fourth edition). Of the 10 studies using assessments of gambling behavior to determine participant eligibility, eight focused on how frequently participants gambled and five focused on the participants’ monetary expenditures on gambling.

Table 2 presents the characteristics of participants and PFIs included in the systematic review and meta-analysis. Participants were an average of 33.56 years old. Most participants identified as men (68%) and as White (60%).

**Table 2:** Characteristics of participants and personalized feedback interventions included in the systematic review and meta-analysis

Study	Total *N*	Mean Age	% Male	% White	PFI Condition	Normative feedback	Psycho-education	Severity feedback	MI?	Therapist-facilitated?
Broussard and Wulfert (2019)	58	38	54	68	PFI	Y	Y	N	Y	Y
Celio and Lisman (2014)	136	19	55	NR	PFI	Y	N	N	N	N
Cunningham et al. (2009)	49	44	47	NR	PFI	Y	N	Y	Y	N
Cunningham et al. (2012)	209	47	53	NR	Full PFI Partial PFI	Y N	Y Y	Y Y	N N	N N
Hopper (2008)	60	21	90	85	PFI	Y	N	N	N	N
Jonsson et al. (2019)	3009	53	81	NR	Phone PFI Letter PFI	N N	Y Y	N N	Y N	Y N
Larimer et al. (2012)	103	21	NR	60	PFI	Y	Y	N	Y	Y
Lostutter (2009)	158	20	70	39	PFI + advice PFI	Y Y	N Y	N N	N N	N N
Luquiens et al. (2016)	557	34	93	NR	Email PFI	Y	N	Y	N	N
Martens et al. (2015)	220	22	60	78	PFI	Y	N	Y	N	N
McAfee et al. (2020)	255	22	62	76	PFI with text messages PFI with education	Y Y	Y Y	N N	Y N	N N
Neighbors et al. (2015)	252	23	60	33	PFI	Y	N	N	N	N
Petry et al. (2008)	140	44	61	64	PFI Brief PFI	Y Y	Y Y	N N	Y N	Y Y
Petry et al. (2009)	96	20	83	82	PFI Brief PFI	Y Y	Y Y	N N	Y N	Y Y
Petry et al. (2016)	135	42	65	28	Brief PFI	Y	Y	N	N	Y
Rosen et al. (2020)	102	32	85	47	Brief PFI	N	Y	N	Y	N
Tabri et al. (2024)	4091	57	62	71	PFI	Y	N	N	N	N
Yokomitsu et al. (2024)	274	45	83	NR	PFI	Y	Y	Y	N	N

*N* no, *NR* not reported, *PFI* personalized feedback intervention, *Y* yes

Across the 18 studies, there were 24 PFI conditions. 75% of the PFIs included normative feedback, 67% included psychoeducation, 25% included feedback on the severity of gambling harm, 33% were supplemented with motivational interviewing, and 33% were facilitated by therapists.

### Outcomes and Assessment Time Points

Table 3 presents each study’s relevant outcomes and assessment time points. Across all 18 studies, there were 9,869 total participants. Most studies (83%) reported gambling expenditure as an outcome, 67% reported gambling frequency, and 67% reported gambling harm. Two studies (12%) reported no gambling outcomes and no studies (0%) reported gambling duration as an outcome, despite recommendations from the Banff Alberta consensus (Walker et al., 2006). Assessment time point weeks ranged from 1 to 96 weeks postintervention. The average timing of the postintervention assessment was 7.47 weeks (*SD* = 5.99) after intervention, and the timing of the longest available follow-up assessment was 29.60 weeks (*SD =* 27.53).

**Table 3:** Relevant study conditions, outcomes, and assessment time points in studies included in the systematic review and meta-analysis

Study	Total *N*	Relevant study conditions (*n*)	Outcomes	Assessment time points (weeks since intervention)
Broussard and Wulfert (2019)	58	PFI (29) Attention control (29)	Frequency Expenditure	2 weeks 4 weeks
Celio and Lisman (2014)	136	PFI (68) Attention control (68)	None	1 week
Cunningham et al. (2009)	49	PFI (24) Assessment only (25)	Expenditure Harm	12 weeks
Cunningham et al. (2012)	209	Full PFI (70) Partial PFI (70) Waitlist (69)	Frequency Expenditure	12 weeks 24 weeks 48 weeks
Hopper (2008)	60	PFI (30) Assessment only (30)	Frequency Expenditure	4 weeks
Jonsson et al. (2019)	3009	Phone PFI (1003) Letter PFI (1003) Assessment only (1003)	Expenditure	14–25 weeks 26–65 weeks
Larimer et al. (2012)	103	MI + PFI (52) Assessment only (51)	Frequency Expenditure Harm	24 weeks
Lostutter (2009)	123	PFI + advice (36) PFI (44) Assessment only (43)	Frequency Expenditure Harm	1 week 4 weeks
Luquiens et al. (2016)	557	Email PFI (293) Waitlist (264)	Frequency Expenditure Harm	6 weeks 12 weeks
Martens et al. (2015)	220	PFI (111) Assessment only (109)	Frequency Expenditure Harm	12 weeks
McAfee et al. (2020)	255	PFI with text messages (86) PFI with education (73) Assessment only (96)	Expenditure Harm	4 weeks 24 weeks
Neighbors et al. (2015)	252	PFI (124) Control feedback (128)	Frequency Expenditure Harm	12 weeks 24 weeks
Petry et al. (2008)	140	MI + PFI (55) Brief PFI (37) Assessment only (48)	Expenditure Harm	6 weeks 36 weeks
Petry et al. (2009)	96	MI + PFI (30) Brief PFI (32) Assessment only (34)	Frequency Expenditure Harm	6 weeks 36 weeks
Petry et al. (2016)	135	Brief PFI (66) Psychoeducation (69)	Frequency Harm	8 weeks 20 weeks 32 weeks 48 weeks 64 weeks 80 weeks 96 weeks
Rosen et al. (2020)	102	PFI (51) Referral only (51)	Frequency Expenditure Harm	4 weeks
Tabri et al. (2024)	4091	PFI (1940) Assessment only (2151)	None	12 weeks
Yokomitsu et al. (2024)	274	PFI (141) Assessment only (133)	Frequency Expenditure Harm	1 week 4 weeks 12 weeks

*MI* motivational intervention, *PFI* personalized feedback intervention. Data points were also extracted from a secondary study by Jonsson et al. (2020)

### Postintervention Outcomes

Table 4 presents all outcomes of PFIs at postintervention. When combining all outcomes (*k* = 15, 44 effect sizes), PFIs demonstrated a small, nonsignificant effect relative to control conditions at postintervention (*g =* −0.06, 95% CI [−0.15, −0.02]). Outcomes were significantly heterogeneous (*Q*(43) = 91.98, *p* <.001), and the range of the 95% PI was modest [−0.35, 0.22]. When examining each outcome separately, the effect size was largest for gambling expenditure (*k* = 13, 18 effect sizes; *g* = −0.09, 95% CI [−0.21, 0.02]), and the effect size was lowest for gambling frequency (*k* = 10, 11 effect sizes; *g* = −0.01, 95% CI [−0.12, 0.11]. Across all outcomes, PFIs were not significantly better at reducing outcomes than control conditions.

**Table 4:** Outcomes of personalized feedback interventions at postintervention and follow-up with confidence intervals and prediction intervals

Postintervention					
Outcome	*k*	# of effect sizes	Hedges’s *g*	95% CI	95% PI
All combined	15	44	−0.06	−0.15, 0.02	−0.35, 0.22
Frequency	10	11	−0.01	−0.12, 0.11	−0.24, 0.23
Expenditure	13	18	−0.09	−0.21, 0.02	−0.36, 0.17
Harm	11	14	−0.07	−0.20, 0.05	−0.34, 0.20
Follow-Up					
Outcome	*k*	# of effect sizes	Hedges’s *g*	95% CI	95% PI
All combined	10	31	−0.10	−0.18, −0.02	−0.37, 0.17
Frequency	6	7	−0.02	−0.17, 0.14	−0.48, 0.45
Expenditure	9	14	−0.12	−0.22, −0.02	−0.37, 0.12
Harm	7	10	−0.16	−0.32, −0.01	−0.33, 0.00

*CI* confidence interval, k number of studies, *PI* prediction interval

Table 5 presents the results of the moderator analyses. Results indicated that studies with PFIs that were therapist facilitated (*g* = −0.18) had significantly better outcomes than studies with PFIs that were not therapist facilitated (*g* = −0.03). Studies with PFIs that incorporated any principles of motivational interviewing (*g* = −0.19) had significantly better outcomes than studies with PFIs that did not incorporate any principles of motivational interviewing (*g =* −0.02). No other variables were found to significantly moderate outcomes of PFIs (*p* <.05).

**Table 5:** Moderator analyses of personalized feedback interventions for gambling at posttreatment with all outcomes combined

Moderator (k)	Hedges’s g	95% CI	Q-value
Sample			1.32
Community sample	−0.02	−0.15, 0.11	
Other sample	−0.10	−0.16, −0.04	
Therapist facilitated			5.24*
Yes	−0.18	−0.29, −0.07	
No	−0.03	−0.09, 0.03	
Motivational interviewing principles			11.55**
Yes	−0.19	−0.27, −0.12	
No	−0.02	−0.09, 0.04	
Normative feedback			0.54
Yes	−0.05	−0.11, 0.01	
No	−0.10	−0.23, 0.02	
Feedback about harm			4.42
Yes	0.00	−0.08, 0.09	
No	−0.12	−0.18, −0.05	
Psychoeducation content			0.27
Yes	−0.05	−0.13, 0.02	
No	−0.09	−0.16, −0.00	
Study eligibility requiring participants to experience gambling harm			0.02
Yes	−0.07	−0.13, −0.00	
No	−0.08	−0.19, 0.04	

*p =.02

**p =.0007

### Follow-Up Outcomes

Table 4 also presents all outcomes of PFIs at follow-up. When combining all outcomes (*k* = 10, 31 effect sizes), PFIs demonstrated a small effect relative to control conditions at follow-up (*g* = −0.10, 95% CI [−0.18, −0.02]). Outcomes were significantly heterogeneous (*Q*(30) = 71.81, *p* <.001) and the range of the 95% PI was modest [−0.37, 0.17]. When examining each outcome separately, the effect size was largest for gambling harm (*k* = 7, 10 effect sizes; *g* = −0.16, 95% CI [−0.32, −0.01]), and the effect size was lowest for gambling frequency (*k* = 6, 7 effect sizes; *g* = −0.02, 95% CI [−0.17, 0.14]). Across all outcomes, PFIs were significantly better in reducing outcomes than control conditions, except for gambling frequency. No moderator analyses were performed on follow-up outcomes due to insufficient statistical power (Higgins & Green, 2008).

A series of sensitivity analyses were conducted to explore the effects of PFIs on outcomes over time in weeks since the intervention (Supplemental Table 3). Analyses were consistent with the postintervention and follow-up assessment, where effect sizes were smallest 1–12 weeks from intervention and largest 13 + weeks after intervention. Similarly, the effects of PFIs were largest for gambling expenditure and harm and smallest for gambling frequency. However, there was limited statistical power to estimate effect sizes at precise time intervals following the receipt of PFIs.

### Risk of Bias Assessment

Table 6 summarizes the risk of bias for all studies based on the Cochrane Risk of Bias 2.0 tool. Only one study (6%) was classified as low risk of bias in all dimensions. However, the individual dimensions of study quality were relatively strong except for the selection of results. More than half of the studies (67%) were rated low risk for their randomization process, 15 studies (83%) were rated low risk for intervention deviations, 11 studies (61%) were rated low bias for missing data, and 14 studies (78%) were rated low risk for outcome measurement. Only three studies (17%) were rated low risk for selection of results due to a lack of preregistration.

**Table 6:** Cochrane risk of bias 2.0 assessment for studies included in the systematic review and Meta-Analysis on personalized feedback interventions for gambling

Study	Randomization Process	Intervention Deviations	Missing Data	Outcome Measurement	Selection of Results
Broussard and Wulfert (2019)	+	+	-	+	?
Celio and Lisman (2014)	?	+	+	+	?
Cunningham et al. (2009)	?	+	-	+	?
Cunningham et al. (2012)	+	+	?	+	-
Hopper (2009)	?	-	-	-	?
Jonsson et al. (2019)	+	+	+	+	?
Larimer et al. (2012)	?	-	?	+	+
Lostutter (2009)	+	+	-	+	?
Luquiens et al. (2016)	+	+	?	+	?
Martens et al. (2015)	+	+	+	+	?
McAfee et al. (2020)	+	-	+	+	?
Neighbors et al. (2015)	?	+	+	+	?
Petry et al. (2008)	+	+	+	+	?
Petry et al. (2009)	+	+	+	+	+
Petry et al. (2016)	+	+	+	-	?
Rosen et al. (2020)	+	+	+	+	?
Tabri et al. (2024)	?	+	+	+	+
Yokomitsu et al. (2024)	+	+	+	+	?

+ = low risk of bias; - = high risk of bias; ? = some concerns

## Discussion

The aim of the current systematic review and meta-analysis was to estimate the effects of PFIs on various gambling outcomes over time in the context of secondary prevention. Using correlated, hierarchical effects meta-analysis, PFIs had nonsignificant, small reductions in outcomes at postintervention (*g* = −0.06, 95% CI [−0.15, 0.02]). The magnitude of this effect size was less than half of what has been found in previous meta-analyses (Peter et al., 2019; Quilty et al., 2019). This difference is probably due to the current meta-analysis’s methodological difference that excluded studies that combined PFIs with cognitive-behavioral interventions, which were included in the two previous meta-analyses. Cognitive-behavioral interventions engender large changes in gambling behavior and gambling harm relative to control conditions (Pfund et al., 2023), and it is likely that including cognitive-behavioral interventions in the previous meta-analysis inflated the magnitude of the effect of PFIs in the short-term.

The nonsignificant, small effect of PFIs on gambling outcomes was, however, consistent with Saxton and colleagues’ (2021) meta-analysis that compared the effectiveness of PFIs across various addictive behaviors. Their results indicated that PFIs had a small, nonsignificant effect on gambling outcomes, and this effect was smaller than the effect of PFIs on alcohol use. While the scope of this study does not allow understanding of why PFIs may be more effective for alcohol than gambling, it is worth noting the heterogeneity in the participant eligibility criteria for studies on PFIs for gambling. In studies on PFIs for gambling, different indicators (i.e., gambling behavior and harm), thresholds (i.e., reaching a certain threshold of monetary expenditures or experiences of harm), and assessments (i.e., Problem Gambling Severity Index, South Oaks Gambling Screen) were used to assess participant eligibility criteria. By contrast, studies on PFIs for alcohol had almost uniformly adopted the criteria for binge drinking (i.e., ≥ 4 standard drinks for women on a single occasion, ≥ 5 standard drinks for men) to determine participant eligibility (Saxton et al., 2021). The heterogeneity in the participant eligibility criteria for studies on PFIs for gambling suggests that there has been little consensus on who is likely to need or benefit from PFIs for gambling. Although there was a nonsignificant, small effect of PFIs for gambling in the short-term, the effect size was higher among studies that had therapists facilitate the PFIs and incorporated motivational interviewing principles. Specifically, the effect size for therapist facilitated PFIs (*g* = −0.18) was six times higher than the effect size for non-therapist facilitated PFIs (*g* = −0.03), and the effect size for PFIs incorporating motivational interviewing (*g* = −0.19) was almost ten times higher than the effect size for PFIs not incorporating motivational interviewing (*g* = −0.02). These results were consistent with Peter and colleagues’ (2019) meta-analysis that found the same two moderators were associated with outcomes of PFIs for gambling. These findings are not surprising given that therapists’ use of motivational interviewing techniques has been positively associated with clients’ language about commitment to change gambling behavior (Hodgins, Ching et al., 2009a).

No other variables were found to significantly moderate PFI outcomes. While it is possible that these variables were not truly moderators of PFI effectiveness, it is equally possible that the moderators selected were not appropriately classified. One example was the participant eligibility criteria moderator, which was defined as the requirement for participants to screen positive for the experience of gambling harm (or not). It is possible that this conceptualization of the variable was inappropriate and other aspects of participant eligibility criteria were more relevant (e.g., reaching a specific threshold of gambling frequency or expenditures). Another example was the sample moderator, which was defined as recruiting participants from the community (or not). Some samples were recruited from substance use treatment centers, probation and parole offices, crowdsourcing websites, and gambling websites (Jonsson et al., 2019; Rosen et al., 2020; Tabri et al., 2024; Yokomitsu et al., 2024), while other samples were recruited from college campuses (Broussard & Wulfert, 2019; Larimer et al., 2012). Future research will be needed to understand whether other aspects of study samples (e.g., college students versus not) were important moderators of outcomes. Other moderators not included in this meta-analysis are also worthy of future studies, including whether participants were actively seeking help for gambling.

The current meta-analysis also provided novel information about the effect of PFIs on different gambling outcomes. Specifically, PFIs consistently appeared to produce the greatest reductions on gambling harm and expenditure, but almost no reductions in gambling frequency compared to no intervention conditions at any time point. Such findings appeared to support that PFIs strike an important balance from a harm reduction framework that may allow individuals to continue engaging in gambling but within safe financial limits. However, it is important to recognize that increased gambling frequency has been consistently associated with greater gambling harm (Currie et al., 2006, 2012), and future studies should understand how to optimize the effects of PFIs on all outcomes, particularly gambling frequency.

Another novel finding was that PFIs were shown to affect gambling outcomes over time. PFIs produced significant, small reductions in gambling behavior and gambling harm at follow up (*g* = −0.10, 95% CI [−0.18, −0.02]). Sensitivity analyses partially supported these results, where the effect of PFIs produced small reductions in gambling behavior and gambling harm at 13–25 weeks after receipt of the PFI (*g* = −0.12, 95% CI [−0.21, −0.03]) and at 26 + weeks (*g* = −0.13, 95% CI [−0.31, 0.05]), suggesting that there may be a “sleeper effect” (Bell et al., 2013) where the magnitude of reductions in outcomes increases over time following receipt of PFIs.

Several limitations should be considered when interpreting the results of the current study. Perhaps the most notable limitation was the lack of studies in the body of literature evaluating the effectiveness of PFIs. The lack of studies in this area required pooling what could be different interventions with different populations, as few studies reported outcomes at follow-up, and the timing of these assessments was variable. These differences were especially evident in the follow-up results where the 95% confidence intervals (CI) for the effects of PFIs on gambling expenditure (*g =* −0.12, 95% [−0.22, −0.02] and gambling harm (*g* = −0.16, 95% CI [−0.32, −0.01] were close to overlapping with zero and suggested a lack of statistical power. Only eight studies included longitudinal assessments of outcomes of any kind, and this limited number of studies prevented a comprehensive understanding of the effect of PFI over time. Furthermore, the lack of standardization for the timing of longitudinal assessments prevented a comprehensive understanding of the effects of PFIs on outcomes at specific numbers of weeks following the receipt of PFIs. Future studies would benefit from adding longitudinal assessments with standardized timings that allow comparison to existing studies.

A second limitation was that the PFIs were compared to inactive control conditions, and active control conditions were not included. The lack of active control conditions prevented understanding of PFI effectiveness relative to expectation/placebo effects, which have been found large in the context of other gambling interventions (Ioannidis et al., 2025). Future studies will need to understand the degree to which PFIs are effective beyond placebo conditions.

A third limitation was that it was not possible to understand what specific components of PFIs were associated with outcomes. Across studies, there was generally a lack of detail about what feedback that participants received (e.g., gambling frequency, expenditure, and/or harm) and how this feedback was presented (e.g., graphically or within text). These details are critical for future studies, especially as it relates to understanding how to optimize the effects of PFIs.

A fourth limitation was that no studies were rated low risk of bias across all dimensions of risk of bias. The presence of bias in existing studies necessitates the design and execution of future randomized controlled trials that use preregistration, appropriate procedures for randomization, adequate descriptions of their interventions, and appropriate methods for handling missing data. Such design features will prove challenging as some countries do not provide adequate funding to execute rigorous trials of methods that prevent and treat gambling harm (Weinstock, 2018).

A fifth limitation was that the current body of literature largely represented White men aged 30 years from North America. This limitation suggests that research on PFIs is needed in other geographic regions where the prevalence of gambling problems is high (e.g., Australasia, Europe, Middle East, and Latin America; Tran et al., 2024) to understand if the results generalize. Furthermore, more diverse samples are needed to represent individuals of different ages, genders, races, sexual orientations, and socioeconomic statuses (Peter et al., 2021).

Despite these limitations, the meta-analysis found that therapist-assisted PFIs and PFIs incorporating motivational interviewing were effective in reducing gambling behavior and harm at postintervention relative to control conditions. Furthermore, PFIs were found to produce clinically meaningful reductions in gambling harm and gambling expenditure at follow-up relative to control conditions. As legalized opportunities for gambling continue to expand across the globe (Ukhova & Volberg, 2025) and concerns about gambling harm heighten (Tran et al., 2024; Wardle et al., 2024), effective interventions will prove necessary to reduce gambling behavior and gambling harm.

## Supplementary Information

Below is the link to the electronic supplementary material.

Supplementary Materil 1 (DOCX. 3.70 MB)
