# Supplementary material for: Personalized Feedback Interventions for Indicated Prevention of Gambling Disorder: A Systematic Review and Meta-Analysis
Source: J Gambl Stud. 2025 Oct 29;41(4):1563–81. doi: 10.1007/s10899-025-10444-5 (PMC12657531; doi:10.1007/s10899-025-10444-5)
Supplement: Supplementary file 1 — Supplementary Materil 1 (DOCX. 3.70 MB) [file 10899_2025_10444_MOESM1_ESM.docx]

**Supplemental Materials for**

**Personalized Feedback Interventions for Indicated Prevention of Gambling Disorder:**

**A Systematic Review and Meta-Analysis**

E. Halle Smith^12^, Bre’Anna L. Free^12^, Meredith K. Ginley^13^, James P. Whelan^12^, & Rory A. Pfund^12^

^1^Tennessee Institute for Gambling Education & Research

^2^ University of Memphis

^3^East Tennessee State University

Supplemental Table 1.

*Search Terms and Boolean Phrases Used in the Systematic Search Strategy for Each Database*

| **Database** | **Search Terms** |
| --- | --- |
| Cochrane Central Register of Controlled Trials | (intervention OR treatment OR therapy) AND gambling |
| Embase | ('intervention':ti OR 'treat*':ti OR 'therapy':ti) AND 'gambl*':ti |
| PsycINFO | TI ("intervention" OR "treat*" OR "therapy") AND TI "gambl*" |
| PubMed | ("intervention"[Title] OR "treat*"[Title] OR "therapy")[Title]) AND ("gambl*"[Title]) |

*Notes*. TI = title

Supplemental Table 2.

*List of Articles Excluded from the Systematic Review and Meta-Analysis at the Full-Text Level*

| **Article** | **Primary reason for exclusion** |
| --- | --- |
| Abbott et al. (2018) | No personalized feedback intervention |
| Auer & Griffiths (2024) | No randomization |
| Auer & Griffiths (2023) | No randomization |
| Auer & Griffiths (2015) | No randomization |
| Baumgartner et al. (2019) | No standard care or no intervention control condition |
| Boudreault et al. (2018) | No personalized feedback intervention |
| Boumparis et al. (2023) | No personalized feedback intervention |
| Bücker et al. (2018) | No personalized feedback intervention |
| Bücker et al. (2021) | No personalized feedback intervention |
| Calado et al. (2020) | No adults |
| Carlbring et al. (2010) | No personalized feedback intervention |
| Carlbring & Smit (2008) | No personalized feedback intervention |
| Casey et al. (2017) | Tertiary prevention study |
| Castrén & Lahti (2014) | No randomization |
| Cunningham et al. (2019) | No personalized feedback intervention |
| Cunningham et al. (2011) | No standard care or no intervention control condition |
| Diaz- Sanahuja et al. (2021) | Study not yet complete |
| Dixon et al. (2016) | No personalized feedback intervention |
| Doiron & Nicki (2007) | No personalized feedback intervention |
| Dowling et al. (2007) | No personalized feedback intervention |
| *DRKS00024841 | Study not yet complete |
| *DRKS00033989 | Study not yet complete |
| Echeburúa et al. (1996) | No personalized feedback intervention |
| Ede et al. (2020) | No personalized feedback intervention |
| Garcia-Palacios et al. (2006) | No personalized feedback intervention |
| Granero et al. (2021) | No randomization |
| Grant et al. (2009) | No personalized feedback intervention |
| Harris & Mazmanian (2016) | No personalized feedback intervention |
| Hodgins et al. (2019) | No standard care or no intervention control condition |
| Hodgins et al. (2009) | Personalized feedback blended with other intervention |
| Hodgins et al. (2001) | Personalized feedback blended with other intervention |
| Humphrey et al. (2020) | No personalized feedback intervention |
| *ISRCTN16969130 | No personalized feedback intervention |
| Jonas et al. (2020) | No personalized feedback intervention |
| *JPRN-UMIN000031759 | Study not yet complete |
| *JPRN-UMIN000046835 | No standard care or no intervention control condition |
| Korman et al. (2008) | No personalized feedback intervention |
| LaBrie et al. (2012) | No personalized feedback intervention |
| Ladouceur et al. (2003) | No personalized feedback intervention |
| Ladouceur et al. (2001) | No personalized feedback intervention |
| Lee & Awosoga (2015) | No personalized feedback intervention |
| Linardatou et al. (2014) | No personalized feedback intervention |
| Maniaci et al. (2018) | No personalized feedback intervention |
| Marceaux & Melville (2011) | No personalized feedback intervention |
| Matsuzaki et al. (2019) | Study not yet complete |
| McIntosh et al. (2016) | No personalized feedback intervention |
| Melville et al. (2004) | No personalized feedback intervention |
| Mide et al. (2023) | No standard care or no intervention control condition |
| Molander et al. (2020) | No randomization |
| Myrseth et al. (2009) | No personalized feedback intervention |
| *NCT02953899 | No personalized feedback intervention |
| *NCT03673800 | No personalized feedback intervention |
| *NCT04064749 | Study not yet complete |
| *NCT05331612 | Personalized feedback blended with other intervention |
| *NCT06011070 | Study not yet complete |
| *NCT06099522 | No personalized feedback intervention |
| *NCT06561139 | No personalized feedback intervention |
| *NCT06642155 | No personalized feedback intervention |
| Oakes et al. (2012) | No randomization |
| Oei et al. (2010) | No personalized feedback intervention |
| Oei et al. (2018) | No personalized feedback intervention |
| Palomäki et al. (2022) | No randomization |
| Petry et al. (2006) | No personalized feedback intervention |
| Rodda et al. (2020) | No personalized feedback intervention |
| Rolvien et al. (2024) | No personalized feedback intervention |
| Savron et al. (2007) | No personalized feedback intervention |
| So et al. (2020) | No personalized feedback intervention |
| Sylvain et al. (1997) | No personalized feedback intervention |
| Tamura et al. (2019) | No randomization |
| Toneatto et al. (2014) | No personalized feedback intervention |
| van der Tempel et al. (2020) | No personalized feedback intervention |
| Wall et al. (2023) | No personalized feedback intervention |
| Wang et al. (2024) | No participants who gambled or experienced harm |
| Wong et al. (2015) | No personalized feedback intervention |

*Note.* * = registered study protocol.

Supplemental Table 3.

*Various Outcomes of Personalized Feedback Interventions for Gambling Over Time*

| 1-12 Weeks Postintervention | | | | | |
| --- | --- | --- | --- | --- | --- |
| Outcome | *k* | # of effect sizes | Hedges’s *g* | 95% CI | 95% PI |
| All combined | 13 | 50 | -0.05 | -0.14, 0.03 | -0.32, 0.22 |
| Frequency | 9 | 14 | -0.01 | -0.13, 0.10 | -0.21, 0.18 |
| Expenditure | 12 | 20 | -0.07 | -0.19, 0.04 | -0.28, 0.13 |
| Harm | 10 | 16 | -0.10 | -0.20, -0.00 | -0.45, 0.24 |
| 13-25 Weeks Postintervention | | | | | |
| Outcome | *k* | # of effect sizes | Hedges’s *g* | 95% CI | 95% PI |
| All combined | 6 | 19 | -0.12 | -0.21, -0.03 | -0.30, 0.06 |
| Frequency | 4 | 5 | -0.03 | -0.20, 0.13 | -0.24, 0.17 |
| Expenditure | 5 | 8 | -0.11 | -0.23, 0.01 | -0.32, 0.10 |
| Harm | 4 | 6 | -0.12 | -0.27, 0.04 | -0.27, 0.04 |
| 26+ Weeks Postintervention | | | | | |
| Outcome | *k* | # of effect sizes | Hedges’s *g* | 95% CI | 95% PI |
| All combined | 5 | 23 | -0.13 | -0.31, 0.05 | -0.32, 0.06 |
| Frequency | 3 | 8 | 0.01 | -0.40, 0.08 | -0.33, 0.35 |
| Expenditure | 2 | 7 | -0.16 | -1.14, 1.17 | -0.39, 0.08 |
| Harm | 4 | 8 | -0.19 | -0.87, 0.49 | -0.70, 0.32 |

*Notes*. CI = confidence interval; *k* = number of studies PI = prediction interval

*
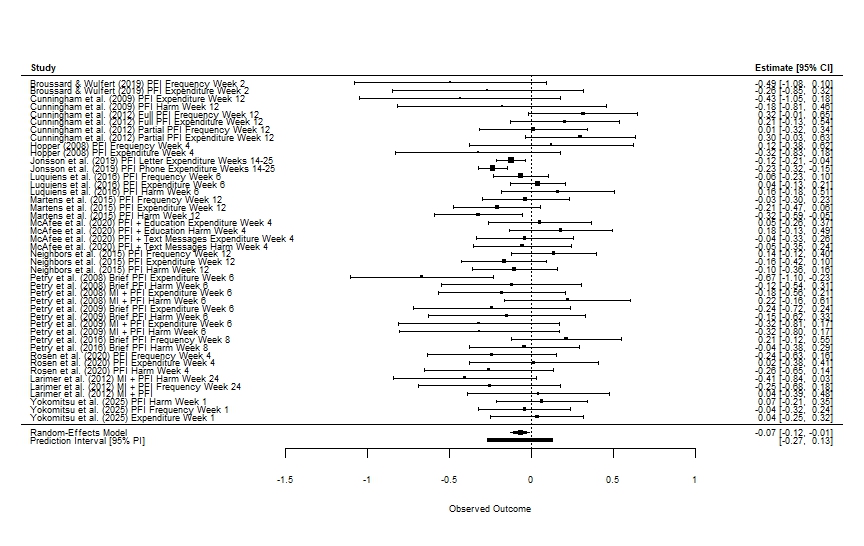

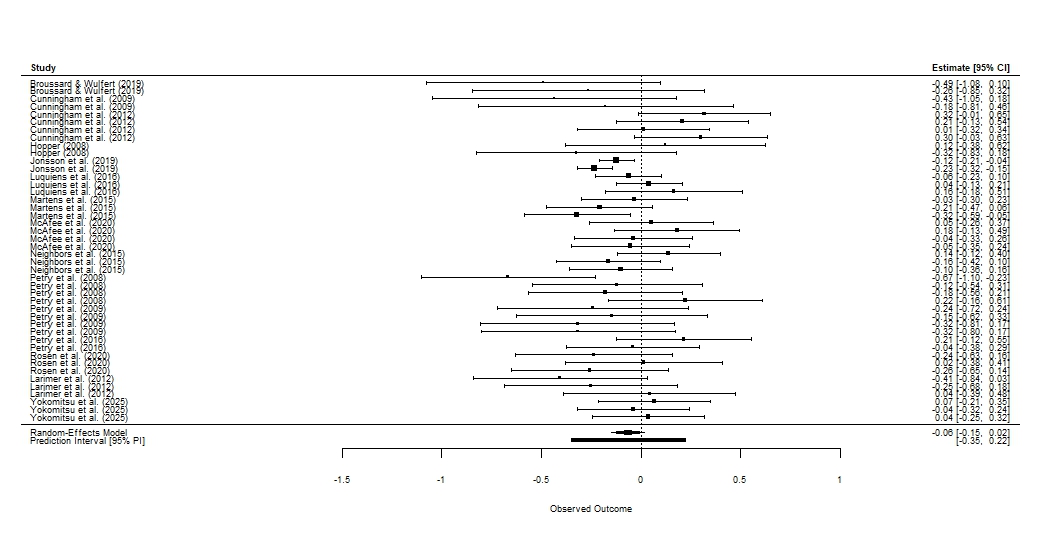
Figure 1.*

Forest Plot of All Outcomes Combined at Postintervention

*
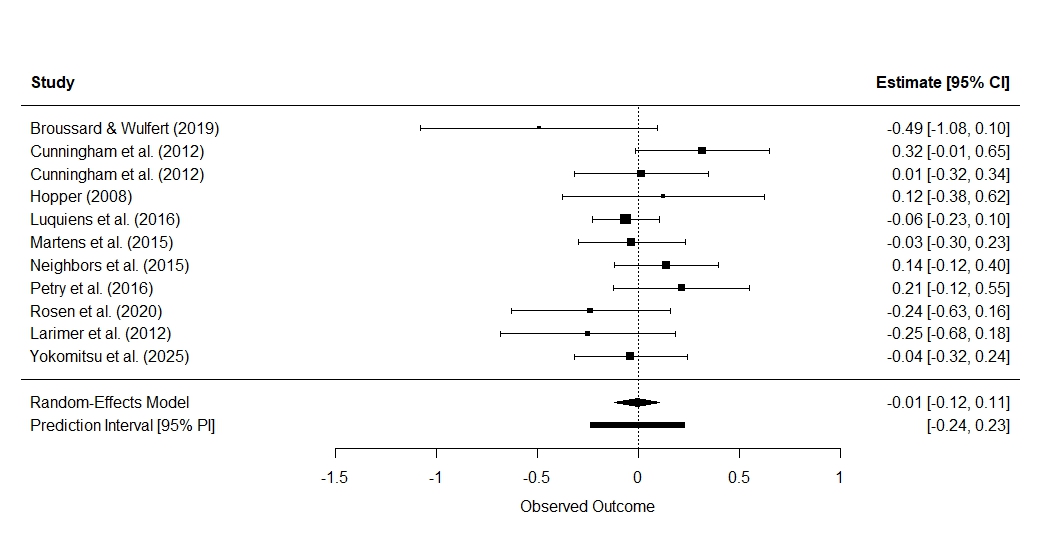

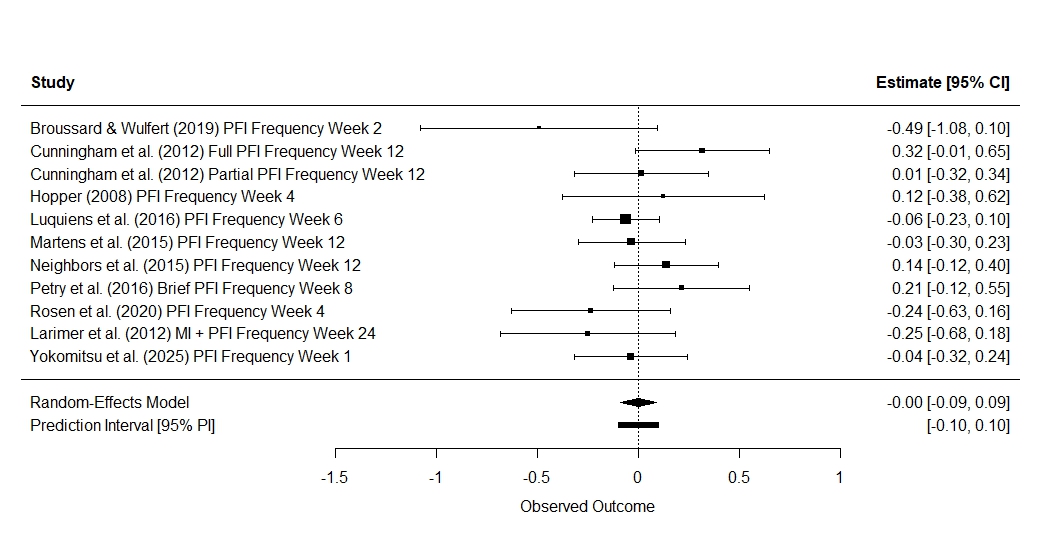
*

*Figure 2.*

Forest Plot of Frequency at Postintervention

*
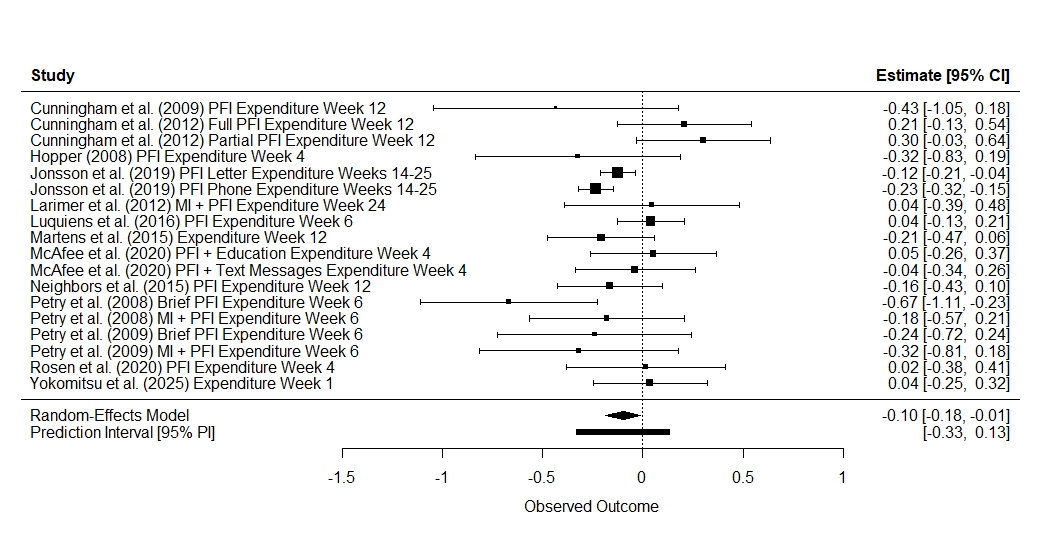

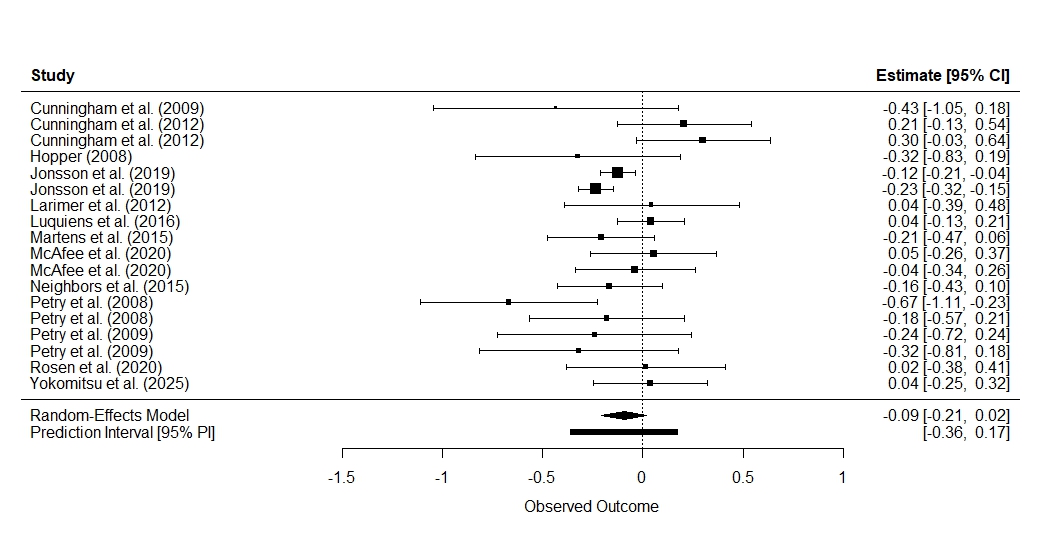
Figure 3.*

Forest Plot of Expenditure at Postintervention

*
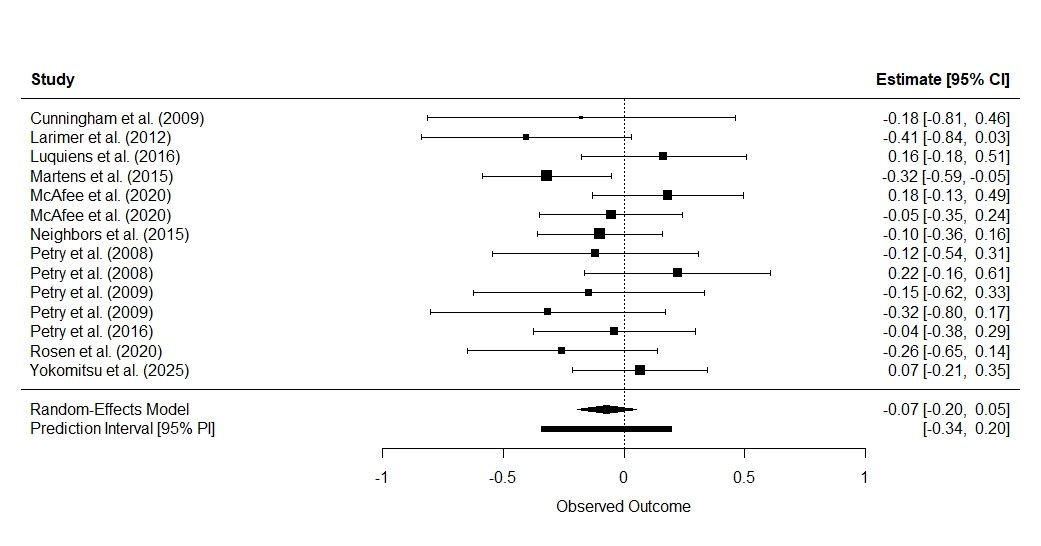

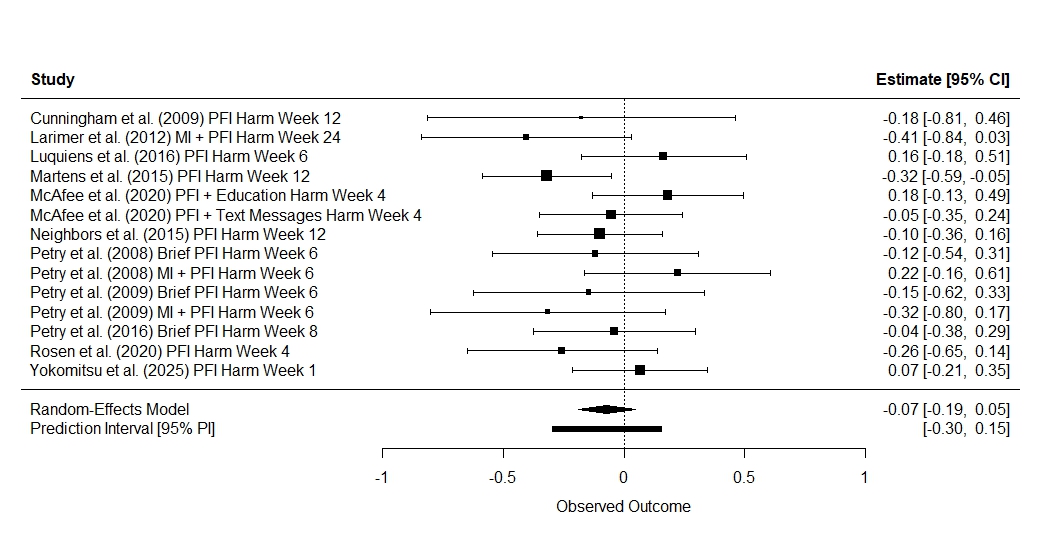
Figure 4.*

Forest Plot of Harm Outcomes at Postintervention

*
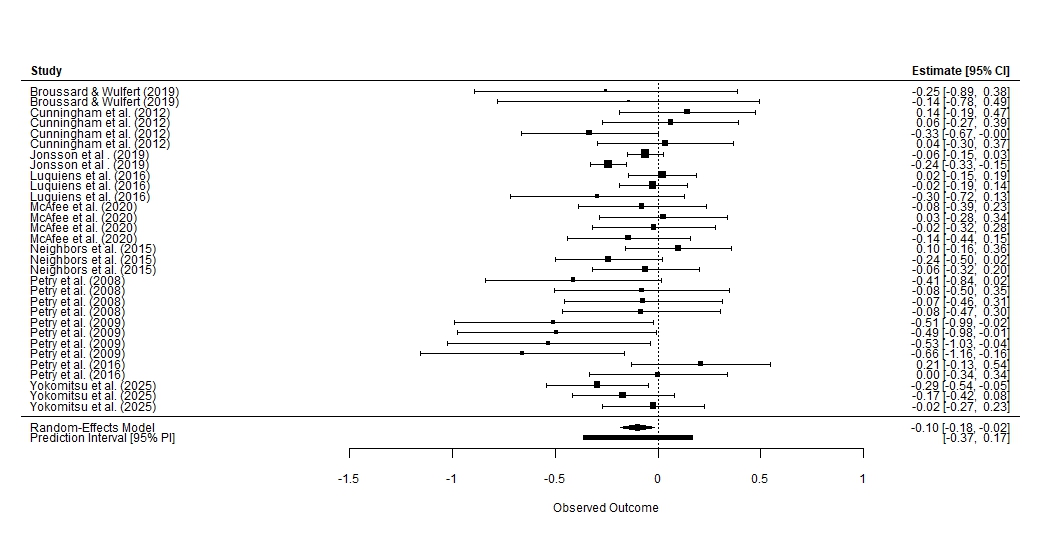
*

*
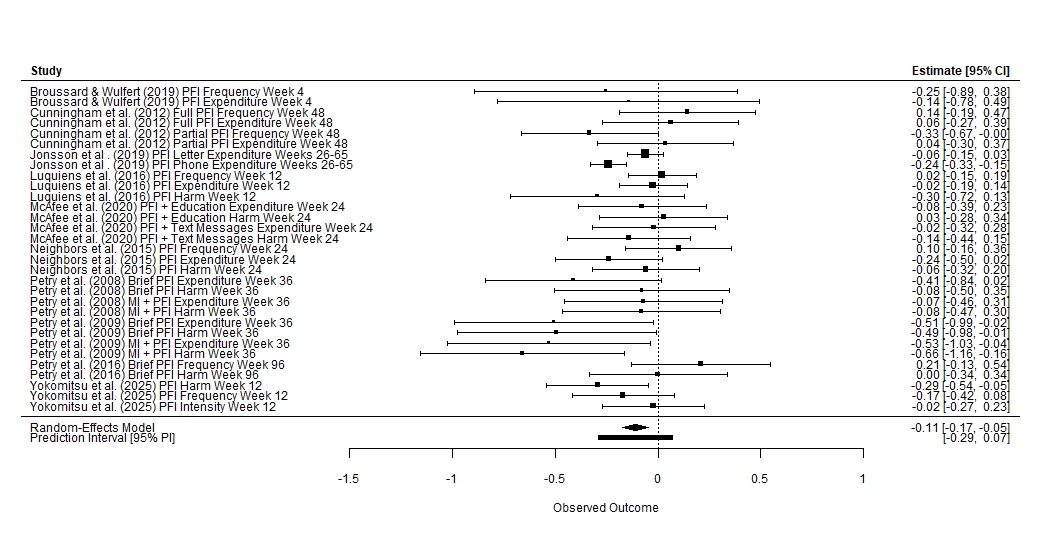
Figure 5.*

Forest Plot of All Outcomes Combined at Follow-Up

*
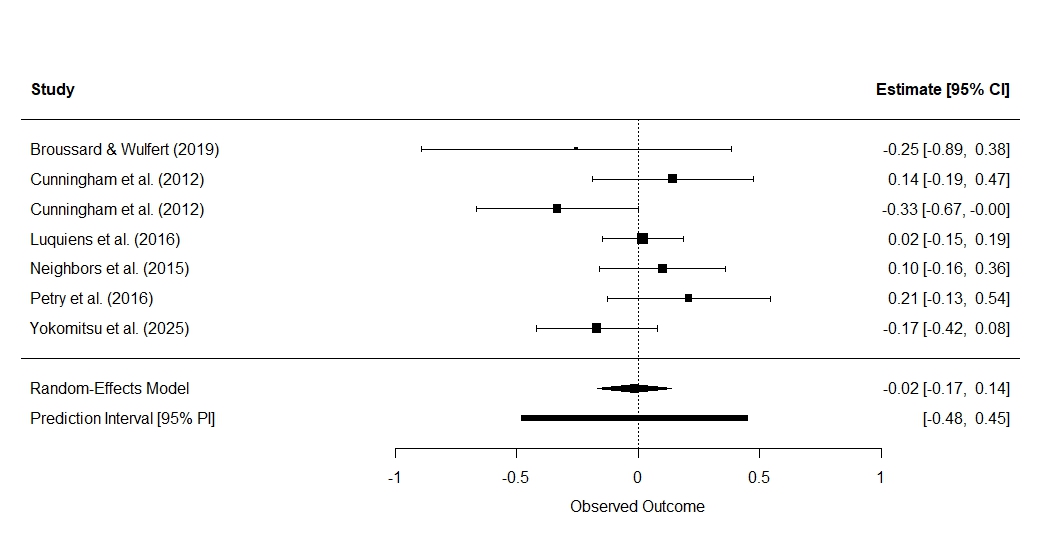
*

*
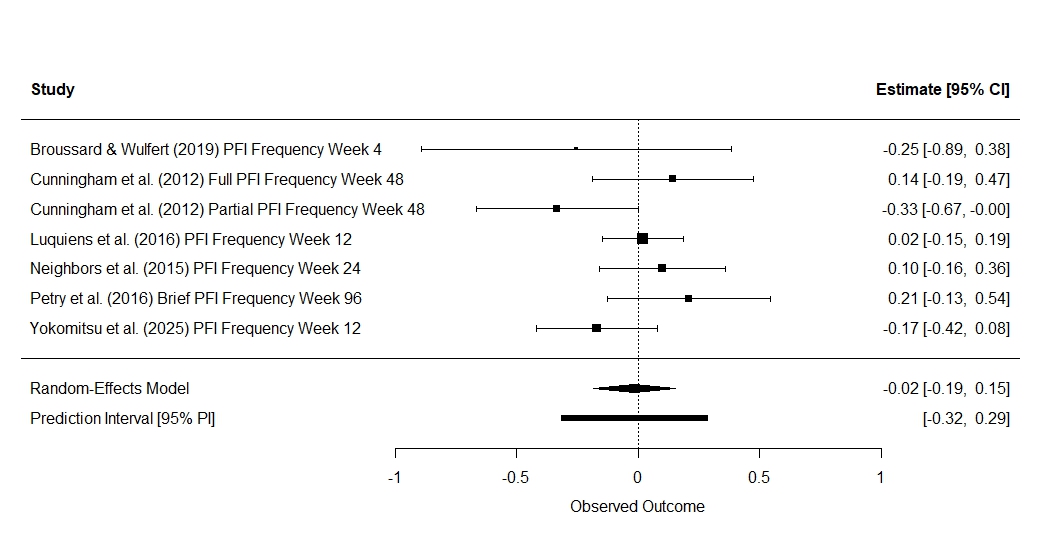
Figure 6.*

Forest Plot of Frequency at Follow-Up

*
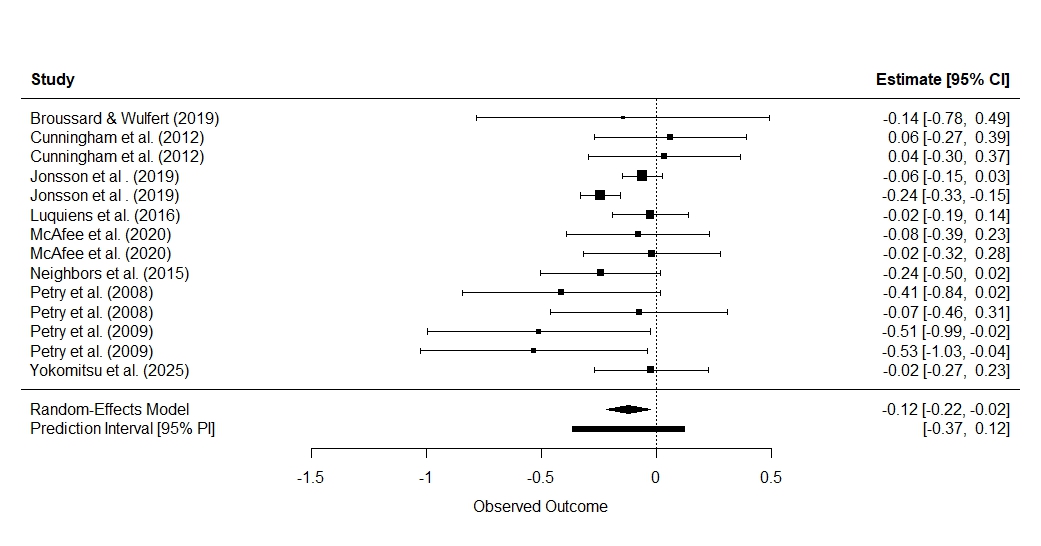
*

*
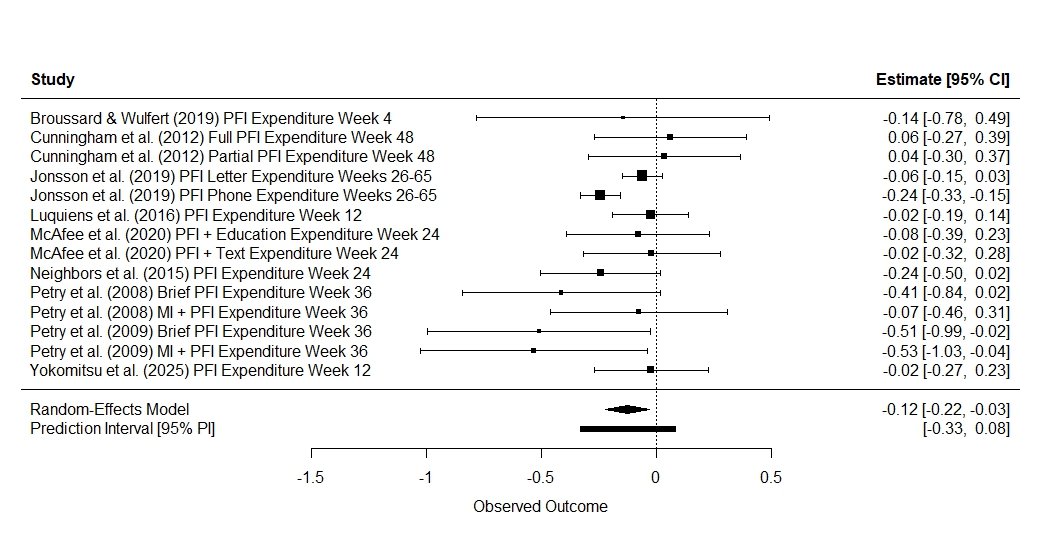
Figure 7.*

Forest Plot of Expenditure at Follow-Up

*
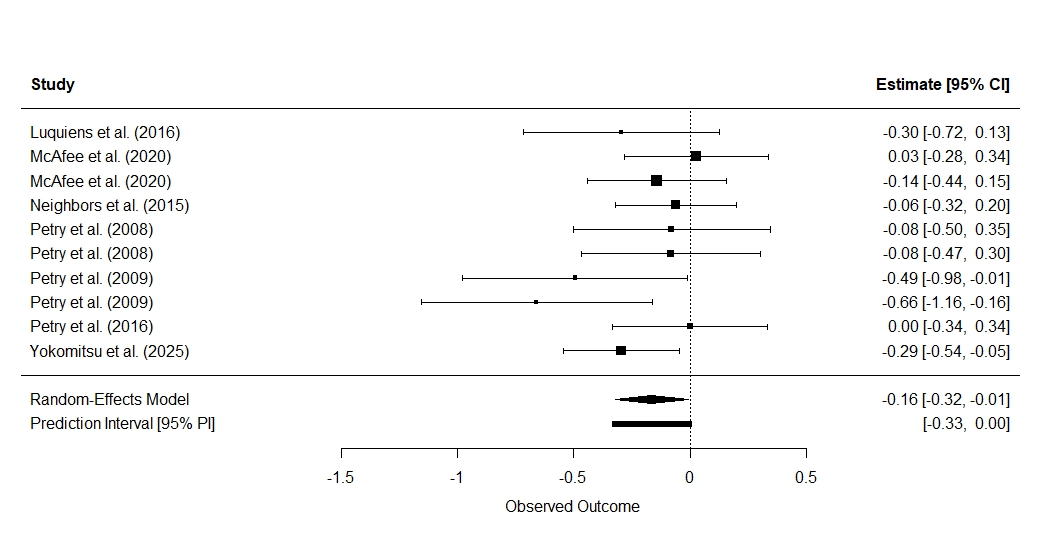
*

*
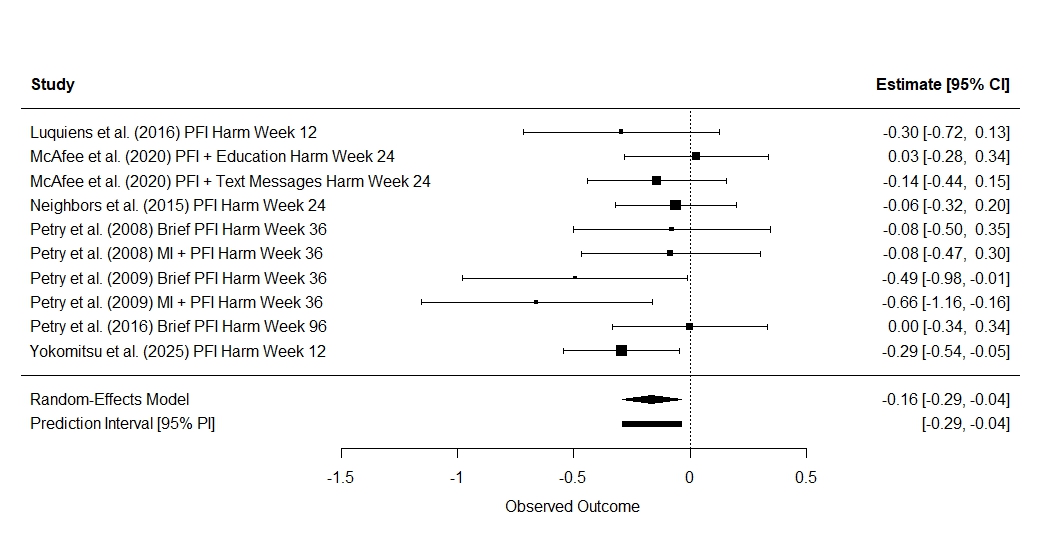
Figure 8.*

Forest Plot of Harm at Follow Up

**Supplemental References**

Abbott, M., Hodgins, D. C., Bellringer, M., Vandal, A. C., Palmer Du Preez, K., Landon, J., ... & Feigin, V. (2018). Brief telephone interventions for problem gambling: A randomized controlled trial. *Addiction, 113*, 883-895. <https://doi.org/10.1111/add.14149>

Auer, M., & Griffiths, M. D. (2024). Nudging online gamblers to withdraw money: The impact of personalized messages on money withdrawal among a sample of real-world online casino players. *Journal of Gambling Studies, 40*, 1227-1244. <https://doi.org/10.1007/s10899-023-10276-1>

Auer, M., & Griffiths, M. D. (2023). The impact of personalized feedback interventions by a gambling operator on subsequent gambling expenditure in a sample of Dutch online gamblers. *Journal of Gambling Studies, 39*, 929-946. <https://doi.org/10.1007/s10899-022-10162-2>

Auer, M. M., & Griffiths, M. D. (2015). The use of personalized behavioral feedback for online gamblers: an empirical study. *Frontiers in Psychology, 6*, 1406. <https://doi.org/10.3389/fpsyg.2015.01406>

Baumgartner, C., Bilevicius, E., Khazaal, Y., Achab, S., Schaaf, S., Wenger, A., ... & Schaub, M. P. (2019). Efficacy of a web-based self-help tool to reduce problem gambling in Switzerland: Study protocol of a two-armed randomised controlled trial. *BMJ Open, 9*(12), e032110. <https://doi.org/10.1136/bmjopen-2019-032110>

Boudreault, C., Giroux, I., Jacques, C., Goulet, A., Simoneau, H., & Ladouceur, R. (2018). Efficacy of a self-help treatment for at-risk and pathological gamblers. *Journal of Gambling Studies, 34*, 561-580. <https://doi.org/10.1007/s10899-017-9717-z>

Boumparis, N., Baumgartner, C., Malischnig, D., Wenger, A., Achab, S., Khazaal, Y., ... & Schaub, M. P. (2023). Effectiveness of a web-based self-help tool to reduce problem gambling: A randomized controlled trial. *Journal of Behavioral Addictions, 12*, 744-757. <https://doi.org/10.1556/2006.2023.00045>

Bücker, L., Bierbrodt, J., Hand, I., Wittekind, C., & Moritz, S. (2018). Effects of a depression-focused internet intervention in slot machine gamblers: A randomized controlled trial. *PloS One, 13*, e0198859. <https://doi.org/10.1371/journal.pone.0198859>

Bücker, L., Gehlenborg, J., Moritz, S., & Westermann, S. (2021). A randomized controlled trial on a self-guided Internet-based intervention for gambling problems. *Scientific Reports, 11*, 13033. <https://doi.org/10.1038/s41598-021-92242-8>

Calado, F., Alexandre, J., Rosenfeld, L., Pereira, R., & Griffiths, M. D. (2020). The efficacy of a gambling prevention program among high-school students. *Journal of Gambling Studies, 36*, 573-595. <https://doi.org/10.1007/s10899-019-09908-2>

Carlbring, P., Jonsson, J., Josephson, H., & Forsberg, L. (2010). Motivational interviewing versus cognitive behavioral group therapy in the treatment of problem and pathological gambling: A randomized controlled trial. *Cognitive Behaviour Therapy, 39*, 92-103. <https://doi.org/10.1080/16506070903190245>

Carlbring, P., & Smit, F. (2008). Randomized trial of internet-delivered self-help with telephone support for pathological gamblers. *Journal of Consulting and Clinical Psychology, 76*, 1090-1094. <https://doi.org/10.1037/a0013603>

Casey, L. M., Oei, T. P., Raylu, N., Horrigan, K., Day, J., Ireland, M., & Clough, B. A. (2017). Internet-based delivery of cognitive behaviour therapy compared to monitoring, feedback and support for problem gambling: A randomised controlled trial. *Journal of Gambling Studies, 33*, 993-1010. <https://doi.org/10.1007/s10899-016-9666-y>

Castrén, S., & Lahti, T. (2014). Game Over – Virtual support for gambling problems: Description of participants and experiences of the treatment method. *Psykologia, 49*, 198-210.

Cunningham, J. A., Godinho, A., & Hodgins, D. C. (2019). Pilot randomized controlled trial of an online intervention for problem gamblers. *Addictive Behaviors Reports, 9*, 100175. <https://doi.org/10.1016/j.abrep.2019.100175>

Cunningham, J. A., Hodgins, D. C., & Toneatto, T. (2011). Pilot study of an Internet-based personalized feedback intervention for problem gamblers. *Journal of Gambling Issues, 26*, 3-10. <http://jgi.camh.net/doi/pdf/10.4309/jgi.2011.26.2>

Diaz-Sanahuja, L., Campos, D., Mira, A., Castilla, D., García-Palacios, A., & Bretón-López, J. M. (2021). Efficacy of an Internet-based psychological intervention for problem gambling and gambling disorder: Study protocol for a randomized controlled trial. *Internet Interventions, 26*, 100466. <https://doi.org/10.1016/j.invent.2021.100466>

Dixon, M. R., Wilson, A. N., & Habib, R. (2016). Neurological evidence of acceptance and commitment therapy effectiveness in college-age gamblers. *Journal of Contextual Behavioral Science, 5*, 80-88. <https://doi.org/10.1016/j.jcbs.2016.04.004>

Doiron, J. P., & Nicki, R. M. (2007). Prevention of pathological gambling: A randomized controlled trial. *Cognitive Behaviour Therapy, 36*, 74-84. <https://doi.org/10.1080/16506070601092966>

Dowling, N., Smith, D., & Thomas, T. (2007). A comparison of individual and group cognitive-behavioural treatment for female pathological gambling. *Behaviour Research and Therapy, 45*, 2192-2202. <https://doi.org/10.1016/j.brat.2006.11.003>

DRKS00024841. *Application of a self-help smartphone app among Turkish-speaking individuals with emotional problems and/or problem gambling behavior: A randomized controlled trial*. <https://drks.de/search/en/trial/DRKS00024841/details>

DRKS00033989. *Effectiveness of the school-based "PROTECT" primary prevention program to reduce Gaming Disorder and problematic internet use in 15 regional centres in Baden-Württemberg, Germany.* <https://drks.de/search/en/trial/DRKS00033989/details>

Echeburúa, E., Báez, C., & Fernández-Montalvo, J. (1996). Comparative effectiveness of three therapeutic modalities in the psychological treatment of pathological gambling: Long-term outcome. *Behavioural and Cognitive Psychotherapy, 24*, 51-72. <https://doi.org/10.1017/S1352465800016830>

Ede, M. O., Omeje, J. C., Ncheke, D. C., Agah, J. J., Chinweuba, N. H., & Amoke, C. V. (2020). Assessment of the effectiveness of group cognitive behavioural therapy in reducing pathological gambling. *Journal of Gambling Studies, 36*, 1325-1339. <https://doi.org/10.1007/s10899-020-09981-y>

Garcia-Palacios, A., de la Vega, N., Botella, C., Baños, R. M., & Quero, S. (2006). Virtual reality in the treatment of pathological gambling. *Cyberpsychology & Behavior, 9*(6), 706-707.

Granero, R., Blaszczynski, A., Fernández-Aranda, F., Gómez-Peña, M., Moragas, L., Aymamí, N., ... & Jiménez-Murcia, S. (2021). Does money control enhance the effectiveness of CBT for gambling disorder? *International Journal of Mental Health and Addiction, 19*, 1045-1064. <https://doi.org/10.1007/s11469-019-00212-z>

Grant, J. E., Donahue, C. B., Odlaug, B. L., Kim, S. W., Miller, M. J., & Petry, N. M. (2009). Imaginal desensitisation plus motivational interviewing for pathological gambling: randomised controlled trial. *The British Journal of Psychiatry, 195*, 266-267. <https://doi.org/10.1192/bjp.bp.108.062414>

Harris, N., & Mazmanian, D. (2016). Cognitive behavioural group therapy for problem gamblers who gamble over the internet: A controlled study. *Journal of Gambling Issues, 33*, 170-188. <http://igi.camh.net/doi/pdf/10.4309/jgi.2016.33.10>

Hodgins, D. C., Cunningham, J. A., Murray, R., & Hagopian, S. (2019). Online self-directed interventions for gambling disorder: Randomized controlled trial. *Journal of Gambling Studies, 35*, 635-651. <https://doi.org/10.1007/s10899-019-09830-7>

Hodgins, D. C., Currie, S. R., Currie, G., & Fick, G. H. (2009). Randomized trial of brief motivational treatments for pathological gamblers: More is not necessarily better. *Journal of Consulting and Clinical Psychology, 77*, 950-960. <https://doi.org/10.1037/a0016318>

Hodgins, D. C., Currie, S. R., & el-Guebaly, N. (2001). Motivational enhancement and self-help treatments for problem gambling. *Journal of Consulting and Clinical Psychology, 69*(1), 50-57. <https://doi.org/10.1037/0022-006X.69.1.50>

Humphrey, G., Chu, J., Dowling, N., Rodda, S., Merkouris, S., Parag, V., ... & Bullen, C. (2020). Manaaki–A cognitive behavioral therapy mobile health app to support people experiencing gambling problems: A randomized control trial protocol. *BMC Public Health, 20*, 1-11. <https://doi.org/10.1186/s12889-020-8304-x>

ISRCTN16969130. *The effectiveness of a cognitive behavioral treatment with virtual reality for problem gambling*. <https://www.isrctn.com/ISRCTN16969130>

Jonas, B., Leuschner, F., Eiling, A., Schoelen, C., Soellner, R., & Tossmann, P. (2020). Web-based intervention and email-counseling for problem gamblers: Results of a randomized controlled trial. *Journal of Gambling Studies, 36*, 1341-1358. <https://doi.org/10.1007/s10899-019-09883-8>

JPRN-UMIN000031759. *Development of a standardized cognitive behavioral therapy (CBT) based outpatient treatment program for gambling disorder, and a multi-center randomized controlled trial (RCT) to evaluate the efficacy of the program*. <https://center6.umin.ac.jp/cgi-open-bin/ctr_e/ctr_view.cgi?recptno=R000036258>

JPRN-UMIN000046835. *An effectiveness trial for support with a chatbot for problem gambling*. <https://center6.umin.ac.jp/cgi-open-bin/icdr_e/ctr_view.cgi?recptno=R000053436>

Korman, L., Collins, J., Littman-Sharp, N., Skinner, W., McMain, S., & Mercado, V. (2008). Randomized control trial of an integrated therapy for comorbid anger and gambling. *Psychotherapy Research, 18*, 454-465. <https://doi.org/10.1080/10503300701858362>

LaBrie, R. A., Peller, A. J., LaPlante, D. A., Bernhard, B., Harper, A., Schrier, T., & Shaffer, H. J. (2012). A brief self-help toolkit intervention for gambling problems: A randomized multisite trial. *American Journal of Orthopsychiatry*, *82*, 278-289. <https://doi.org/10.1111/j.1939-0025.2012.01157.x>

Ladouceur, R., Sylvain, C., Boutin, C., Lachance, S., Doucet, C., & Leblond, J. (2003). Group therapy for pathological gamblers: A cognitive approach. *Behaviour Research and Therapy*, *41*, 587-596. <https://doi.org/10.1016/S0005-7967(02)00036-0>

Ladouceur, R., Sylvain, C., Boutin, C., Lachance, S., Doucet, C., Leblond, J., & Jacques, C. (2001). Cognitive treatment of pathological gambling. *The Journal of Nervous and Mental Disease, 189*, 774-780. <https://doi.org/10.1097/00005053-200111000-00007>

Lee, B. K., & Awosoga, O. (2015). Congruence couple therapy for pathological gambling: A pilot randomized controlled trial. *Journal of Gambling Studies, 31*, 1047-1068. <https://doi.org/10.1007/s10899-014-9464-3>

Linardatou, C., Parios, A., Varvogli, L., Chrousos, G., & Darviri, C. (2014). An 8-week stress management program in pathological gamblers: A pilot randomized controlled trial. *Journal of Psychiatric Research, 56*, 137-143. <https://doi.org/10.1016/j.jpsychires.2014.05.013>

Maniaci, G., La Cascia, C., Ferraro, L., Picone, F., Sideli, L., Seminerio, F., & Cannizzaro, C. (2018). The efficacy of a functional therapy program for gambling disorder: A pilot study. *Acta Medica Mediterranea, 34*, 1447-1452. <https://doi.org/10.19193/0393-6384_2018_5_220>

Marceaux, J. C., & Melville, C. L. (2011). Twelve-step facilitated versus mapping-enhanced cognitive-behavioral therapy for pathological gambling: A controlled study. *Journal of Gambling Studies, 27*, 171-190. <https://doi.org/10.1007/s10899-010-9196-y>

Matsuzaki, T., Matsushita, S., Nishimura, K., Furuno, S., Okada, H., & Higuchi, S. (2019). Effectiveness of CBT-based outpatient treatment program for gambling disorder: multi-study site randomized control trial in Japan. *Journal of Behavioral Addictions, 8*, 68.

McIntosh, C. C., Crino, R. D., & O’Neill, K. (2016). Treating problem gambling samples with cognitive behavioural therapy and mindfulness-based interventions: A clinical trial. *Journal of Gambling Studies, 32*, 1305-1325. <https://doi.org/10.1007/s10899-016-9602-1>

Melville, C. L., Davis, C. S., Matzenbacher, D. L., & Clayborne, J. (2004). Node-link-mapping-enhanced group treatment for pathological gambling. *Addictive Behaviors, 29*, 73-87. <https://doi.org/10.1016/S0306-4603(03)00091-1>

Mide, M., Mattiasson, J., Norlin, D., Sehlin, H., Rasmusson, J., Ljung, S., ... & Gordh, A. S. (2023). Internet-delivered therapist-assisted cognitive behavioral therapy for gambling disorder: a randomized controlled trial. *Frontiers in Psychiatry, 14*, 1243826. <https://doi.org/10.3389/fpsyt.2023.1243826>

Molander, O., Lindner, P., Ramnerö, J., Bjureberg, J., Carlbring, P., & Berman, A. H. (2020). Internet-based cognitive behavior therapy for problem gambling in routine care: Protocol for a non-randomized pilot and feasibility trial. *Pilot and Feasibility Studies, 6*, 1-11. <https://doi.org/10.1186/s40814-020-00647-5>

Myrseth, H., Litlerè, I., Støylen, I. J., & Pallesen, S. (2009). A controlled study of the effect of cognitive–behavioural group therapy for pathological gamblers. *Nordic Journal of Psychiatry, 63*, 22-31. <https://doi.org/10.1080/08039480802055139>

NCT02953899. *Contingency management as an adjunct treatment for rural and remote disordered gamblers*. <https://clinicaltrials.gov/study/NCT02953899>

NCT03673800. *Cognitive control training in online problem gambling*. <https://clinicaltrials.gov/study/NCT03673800>

NCT04064749. *Developing and testing a brief intervention for problem gambling in credit counseling*. <https://clinicaltrials.gov/study/NCT04064749>

NCT05331612. *Blended group CBT protocol for problem gambling and gambling disorder combining group sessions and online modules*. <https://clinicaltrials.gov/study/NCT05331612>

NCT06011070. *Pilot randomised trial of a brief online personalised feedback intervention for gambling harm*. <https://clinicaltrials.gov/study/NCT06011070>

NCT06099522. *Internet-CBT for gambling disorder - A randomized controlled trial*. <https://clinicaltrials.gov/study/NCT06099522>

NCT06561139. *Mindfulness as an add-on intervention in treatment for problem gambling in clinical settings in Sweden*. <https://clinicaltrials.gov/study/NCT06561139>

NCT06642155. *Theory-based intervention for promoting responsible gambling among college students*. <https://clinicaltrials.gov/study/NCT06642155>

Oakes, J., Gardiner, P., McLaughlin, K., & Battersby, M. (2012). A pilot group cognitive behavioural therapy program for problem gamblers in a rural Australian setting. *International Journal of Mental Health and Addiction, 10*, 490-500. <https://doi.org/10.1007/s11469-010-9294-1>

Oei, T. P., Raylu, N., & Casey, L. M. (2010). Effectiveness of group and individual formats of a combined motivational interviewing and cognitive behavioral treatment program for problem gambling: A randomized controlled trial. *Behavioural and Cognitive Psychotherapy, 38*, 233-238. <https://doi.org/10.1017/S1352465809990701>

Oei, T. P. S., Raylu, N., & Lai, W. W. (2018). Effectiveness of a self help cognitive behavioural treatment program for problem gamblers: A randomised controlled trial. *Journal of Gambling Studies, 34*, 581-595. <https://doi.org/10.1007/s10899-017-9723-1>

Palomäki, J., Heiskanen, M., & Castrén, S. (2022). Online 8-week cognitive therapy for problem gamblers: The moderating effects of depression symptoms and perceived financial control. *Journal of Behavioral Addictions, 11*, 75-87. <https://doi.org/10.1556/2006.2021.00091>

Petry, N. M., Ammerman, Y., Bohl, J., Doersch, A., Gay, H., Kadden, R., ... & Steinberg, K. (2006). Cognitive-behavioral therapy for pathological gamblers. *Journal of Consulting and Clinical Psychology, 74*, 555-567. <https://doi.org/10.1037/0022-006X.74.3.555>

Rodda, S. N., Bagot, K. L., Manning, V., & Lubman, D. I. (2020). An exploratory RCT to support gamblers’ intentions to stick to monetary limits: A brief intervention using action and coping planning. *Journal of Gambling Studies, 36*, 387-404. <https://doi.org/10.1007/s10899-019-09873-w>

Rolvien, L., Buddeberg, L., Gehlenborg, J., Borsutzky, S., & Moritz, S. (2024). A Self-Guided Internet-Based Intervention for the Reduction of Gambling Symptoms: A Randomized Clinical Trial. *JAMA Network Open, 7*(6), e2417282-e2417282. <https://doi.org/10.1001/jamanetworkopen.2024.17282>

Savron, G., De Luca, R., & Pitti, P. (2007). Group therapy with pathological gamblers: Results after 6, 12 and 18 months of treatment. *Rivista Di Psichiatria, 42*, 189-204.

So, R., Furukawa, T. A., Matsushita, S., Baba, T., Matsuzaki, T., Furuno, S., ... & Higuchi, S. (2020). Unguided chatbot-delivered cognitive behavioural intervention for problem gamblers through messaging app: A randomised controlled trial. Journal of *Gambling Studies, 36*, 1391-1407. <https://doi.org/10.1007/s10899-020-09935-4>

Sylvain, C., Ladouceur, R., & Boisvert, J. M. (1997). Cognitive and behavioral treatment of pathological gambling: A controlled study. *Journal of Consulting and Clinical Psychology*, *65*, 727-732. <https://doi.org/10.1037/0022-006x.65.5.727>

Tamura, K. (2019). Treatment program in prison for pathological gamblers. *Journal of Behavioral Addictions, 8*, 87-88.

Toneatto, T., Pillai, S., & Courtice, E. L. (2014). Mindfulness-enhanced cognitive behavior therapy for problem gambling: A controlled pilot study. *International Journal of Mental Health and Addiction, 12*, 197-205. <https://doi.org/10.1007/s11469-014-9481-6>

van der Tempel, J., McDermott, K., Niepage, M., Afifi, T. O., McMain, S., Jindani, F., ... & Zack, M. (2020). Examining the effects of mindfulness practice and trait mindfulness on gambling symptoms in women with gambling disorder: A feasibility study. *International Gambling Studies, 20*(1), 114-134. <https://doi.org/10.1080/14459795.2019.1686766>

Wall, H., Magnusson, K., Hellner, C., Andersson, G., Jayaram-Lindström, N., & Rosendahl, I. (2023). The evaluation of a brief ICBT program with therapist support for individuals with gambling problems in the context of a gambling helpline: A randomized pilot trial. *Pilot and Feasibility Studies, 9*, 26. <https://doi.org/10.1186/s40814-023-01257-7>

Wang, Y., Zhao, L., Wan, K., & Zeng, X. (2024). Reducing problematic loot-box use with self-control intervention: A randomized controlled trial. *International Journal of Mental Health and Addiction*, 1-21. <https://doi.org/10.1007/s11469-024-01413-x>

Wong, D. F. K., Chung, C. L. P., Wu, J., Tang, J., & Lau, P. (2015). A preliminary study of an integrated and culturally attuned cognitive behavioral group treatment for Chinese problem gamblers in Hong Kong. *Journal of Gambling Studies, 31*, 1015-1027. <https://doi.org/10.1007/s10899-014-9457-2>

| **Section and Topic** | **Item #** | **Checklist item** | **Reported (Yes/No)** |
| --- | --- | --- | --- |
| **TITLE** | | |  |
| Title | 1 | Identify the report as a systematic review. | Yes |
| **BACKGROUND** | | |  |
| Objectives | 2 | Provide an explicit statement of the main objective(s) or question(s) the review addresses. | Yes |
| **METHODS** | | |  |
| Eligibility criteria | 3 | Specify the inclusion and exclusion criteria for the review. | Yes |
| Information sources | 4 | Specify the information sources (e.g. databases, registers) used to identify studies and the date when each was last searched. | Yes |
| Risk of bias | 5 | Specify the methods used to assess risk of bias in the included studies. | Yes |
| Synthesis of results | 6 | Specify the methods used to present and synthesise results. | Yes |
| **RESULTS** | | |  |
| Included studies | 7 | Give the total number of included studies and participants and summarise relevant characteristics of studies. | Yes |
| Synthesis of results | 8 | Present results for main outcomes, preferably indicating the number of included studies and participants for each. If meta-analysis was done, report the summary estimate and confidence/credible interval. If comparing groups, indicate the direction of the effect (i.e. which group is favoured). | Yes |
| **DISCUSSION** | | |  |
| Limitations of evidence | 9 | Provide a brief summary of the limitations of the evidence included in the review (e.g. study risk of bias, inconsistency and imprecision). | Yes |
| Interpretation | 10 | Provide a general interpretation of the results and important implications. | Yes |
| **OTHER** | | |  |
| Funding | 11 | Specify the primary source of funding for the review. | Yes |
| Registration | 12 | Provide the register name and registration number. | Yes |

| **Section and Topic** | **Item #** | **Checklist item** | **Location where item is reported** |
| --- | --- | --- | --- |
| **TITLE** | | |  |
| Title | 1 | Identify the report as a systematic review. | Page 1 |
| **ABSTRACT** | | |  |
| Abstract | 2 | See the PRISMA 2020 for Abstracts checklist. | Page 2 |
| **INTRODUCTION** | | |  |
| Rationale | 3 | Describe the rationale for the review in the context of existing knowledge. | Pages 3-5 |
| Objectives | 4 | Provide an explicit statement of the objective(s) or question(s) the review addresses. | Page 5 |
| **METHODS** | | |  |
| Eligibility criteria | 5 | Specify the inclusion and exclusion criteria for the review and how studies were grouped for the syntheses. | Pages 6-7 |
| Information sources | 6 | Specify all databases, registers, websites, organisations, reference lists and other sources searched or consulted to identify studies. Specify the date when each source was last searched or consulted. | Page 6, Supplemental Table 1 |
| Search strategy | 7 | Present the full search strategies for all databases, registers and websites, including any filters and limits used. | Page 6, Supplemental Table 1 |
| Selection process | 8 | Specify the methods used to decide whether a study met the inclusion criteria of the review, including how many reviewers screened each record and each report retrieved, whether they worked independently, and if applicable, details of automation tools used in the process. | Pages 7-8 |
| Data collection process | 9 | Specify the methods used to collect data from reports, including how many reviewers collected data from each report, whether they worked independently, any processes for obtaining or confirming data from study investigators, and if applicable, details of automation tools used in the process. | Pages 7-8 |
| Data items | 10a | List and define all outcomes for which data were sought. Specify whether all results that were compatible with each outcome domain in each study were sought (e.g. for all measures, time points, analyses), and if not, the methods used to decide which results to collect. | Page 7 |
|  | 10b | List and define all other variables for which data were sought (e.g. participant and intervention characteristics, funding sources). Describe any assumptions made about any missing or unclear information. | Pages 7-8 |
| Study risk of bias assessment | 11 | Specify the methods used to assess risk of bias in the included studies, including details of the tool(s) used, how many reviewers assessed each study and whether they worked independently, and if applicable, details of automation tools used in the process. | Page 8 |
| Effect measures | 12 | Specify for each outcome the effect measure(s) (e.g. risk ratio, mean difference) used in the synthesis or presentation of results. | Pages 8-9 |
| Synthesis methods | 13a | Describe the processes used to decide which studies were eligible for each synthesis (e.g. tabulating the study intervention characteristics and comparing against the planned groups for each synthesis (item #5)). | Page 8 |
|  | 13b | Describe any methods required to prepare the data for presentation or synthesis, such as handling of missing summary statistics, or data conversions. | Page 9 |
|  | 13c | Describe any methods used to tabulate or visually display results of individual studies and syntheses. | N/A |
|  | 13d | Describe any methods used to synthesize results and provide a rationale for the choice(s). If meta-analysis was performed, describe the model(s), method(s) to identify the presence and extent of statistical heterogeneity, and software package(s) used. | Pages 8-9 |
|  | 13e | Describe any methods used to explore possible causes of heterogeneity among study results (e.g. subgroup analysis, meta-regression). | Page 9 |
|  | 13f | Describe any sensitivity analyses conducted to assess robustness of the synthesized results. | Page 12 |
| Reporting bias assessment | 14 | Describe any methods used to assess risk of bias due to missing results in a synthesis (arising from reporting biases). | Page 8 |
| Certainty assessment | 15 | Describe any methods used to assess certainty (or confidence) in the body of evidence for an outcome. | Page 9 |
| **RESULTS** | | |  |
| Study selection | 16a | Describe the results of the search and selection process, from the number of records identified in the search to the number of studies included in the review, ideally using a flow diagram. | Page 9; Figure 1 |
|  | 16b | Cite studies that might appear to meet the inclusion criteria, but which were excluded, and explain why they were excluded. | Supplemental Table 2 |
| Study characteristics | 17 | Cite each included study and present its characteristics. | Pages 9-11; Tables 1-3 |
| Risk of bias in studies | 18 | Present assessments of risk of bias for each included study. | Table 6 |
| Results of individual studies | 19 | For all outcomes, present, for each study: (a) summary statistics for each group (where appropriate) and (b) an effect estimate and its precision (e.g. confidence/credible interval), ideally using structured tables or plots. | Supplemental Figures 1-8 |
| Results of syntheses | 20a | For each synthesis, briefly summarise the characteristics and risk of bias among contributing studies. | Pages 12-13; Table 6 |
|  | 20b | Present results of all statistical syntheses conducted. If meta-analysis was done, present for each the summary estimate and its precision (e.g. confidence/credible interval) and measures of statistical heterogeneity. If comparing groups, describe the direction of the effect. | Pages 11-12; Table 4 |
|  | 20c | Present results of all investigations of possible causes of heterogeneity among study results. | Pages 11-12; Table 5 |
|  | 20d | Present results of all sensitivity analyses conducted to assess the robustness of the synthesized results. | Page 12; Supplemental Table 3 |
| Reporting biases | 21 | Present assessments of risk of bias due to missing results (arising from reporting biases) for each synthesis assessed. | Pages 12-13; Table 6 |
| Certainty of evidence | 22 | Present assessments of certainty (or confidence) in the body of evidence for each outcome assessed. | Pages 11-12; Table 4 |
| **DISCUSSION** | | |  |
| Discussion | 23a | Provide a general interpretation of the results in the context of other evidence. | Pages 13-18 |
|  | 23b | Discuss any limitations of the evidence included in the review. | Pages 16-17 |
|  | 23c | Discuss any limitations of the review processes used. | Pages 16-17 |
|  | 23d | Discuss implications of the results for practice, policy, and future research. | Pages 13-18 |
| **OTHER INFORMATION** | | |  |
| Registration and protocol | 24a | Provide registration information for the review, including register name and registration number, or state that the review was not registered. | Abstract; Page 5 |
|  | 24b | Indicate where the review protocol can be accessed, or state that a protocol was not prepared. | N/A |
|  | 24c | Describe and explain any amendments to information provided at registration or in the protocol. | Pages 5-6 |
| Support | 25 | Describe sources of financial or non-financial support for the review, and the role of the funders or sponsors in the review. | Page 1 |
| Competing interests | 26 | Declare any competing interests of review authors. | Page 1 |
| Availability of data, code and other materials | 27 | Report which of the following are publicly available and where they can be found: template data collection forms; data extracted from included studies; data used for all analyses; analytic code; any other materials used in the review. | N/A |
